# Efficacy of Transcendental Meditation to Reduce Stress Among Health Care Workers: A Randomized Clinical Trial

**DOI:** 10.1001/jamanetworkopen.2022.31917

**Published:** 2022-09-19

**Authors:** Sangeeta P. Joshi, An-Kwok Ian Wong, Amanda Brucker, Taylor A. Ardito, Shein-Chung Chow, Sandeep Vaishnavi, Patty J. Lee

**Affiliations:** 1 Division of Pulmonary, Allergy, and Critical Care Medicine, Duke University School of Medicine, Durham, North Carolina; 2 Section of Pulmonary and Critical Care, Durham Veterans Administration Medical Center, Durham, North Carolina; 3 Department of Biostatistics and Bioinformatics, Duke University School of Medicine, Durham, North Carolina; 4 Mindpath Health, Raleigh, North Carolina

## Abstract

**Importance:**

Health care workers (HCWs) have been experiencing substantial stress and burnout, and evidence-based mitigation strategies are needed. Transcendental Meditation (TM) is a mantra meditation practice with potential efficacy in reducing stress.

**Objective:**

To assess the efficacy of TM practice in reducing stress among HCWs over a 3-month period.

**Design, Setting, and Participants:**

This single-center open-label randomized clinical trial was conducted among HCWs at an academic medical center from November 19, 2020, to August 31, 2021. Inclusion criteria comprised a score of 6 points or greater on the Subjective Units of Distress Scale and an increase of 5% or greater in baseline heart rate or an increase of 33% or greater in galvanic skin response after exposure to a stressful script. Exclusion criteria included the use of antipsychotic or β blocker medications, current suicidal ideation, or previous TM training. Of 213 HCWs who participated in prescreening, 95 attended in-person visits, resulting in 80 eligible participants who were randomized to receive a TM intervention (TM group) or usual treatment (control group).

**Interventions:**

The TM group practiced TM for 20 minutes twice daily over a 3-month period. The control group received usual treatment, which consisted of access to wellness resources.

**Main Outcomes and Measures:**

The primary outcome was change in acute psychological distress measured by the Global Severity Index. Secondary outcomes included changes in burnout (measured by the Maslach Burnout Inventory), insomnia (measured by the Insomnia Severity Index), and anxiety (measured by the Generalized Anxiety Disorder-7 scale).

**Results:**

Among 80 participants, 66 (82.5%) were women, with a mean (SD) age of 40 (11) years. One participant (1.3%) was American Indian or Alaska Native, 5 (6.3%) were Asian, 12 (15.0%) were Black, 59 (73.8%) were White, and 3 (3.8%) were of unknown or unreported race; 4 participants (5.0%) were Hispanic, and 76 (95.0%) were non-Hispanic. A total of 41 participants were randomized to the TM group, and 39 were randomized to the control group. Participants in the TM group did not show a statistically significant decrease in psychological distress on the Global Severity Index compared with those in the control group (−5.6 points vs −3.8 points; between-group difference, −1.8 points; 95% CI, −4.2 to 0.6 points; P = .13). Compared with the control group, the TM group had significantly greater reductions in the secondary end points of emotional exhaustion (Maslach Burnout Inventory subscore: −8.0 points vs −2.6 points; between-group difference, −5.4 points; 95% CI, −9.2 to −1.6 points; P = .006), insomnia (Insomnia Severity Scale score: −4.1 points vs −1.9 points; between-group difference, −2.2 points; 95% CI, −4.4 to 0 points; P = .05), and anxiety (Generalized Anxiety Disorder-7 score: −3.1 points vs −0.9 points; between-group difference, −2.2 points; 95% CI, −3.8 to −0.5; P = .01) at 3 months. A total of 38 participants (92.7%) in the TM group adhered to home practice.

**Conclusions and Relevance:**

In this randomized clinical trial, TM practice among HCWs over a 3-month period did not result in a statistically significant reduction in the primary outcome of acute psychological distress compared with usual treatment but significantly improved the secondary outcomes of burnout, anxiety, and insomnia. These findings suggest that TM may be a safe and effective strategy to alleviate chronic stress among HCWs.

**Trial Registration:**

ClinicalTrials.gov identifier: NCT04632368

## Key Points

**Question:**

Does the practice of Transcendental Meditation (TM) reduce stress among health care workers (HCWs)?

**Findings:**

This randomized clinical trial of 80 HCWs showed that TM practice over 3 months reduced psychological distress scores (primary outcome) on the Global Severity Index by 5.6 points, but this decrease was not significantly different from the reduction of 3.8 points observed in the control group. The practice of TM reduced burnout scores (a secondary outcome) by 5.4 points, representing a statistically significant reduction compared with usual treatment.

**Meaning:**

This study found that TM practice did not significantly decrease acute distress compared with usual treatment; however, TM significantly reduced chronic stress, particularly burnout, suggesting that TM could be an effective strategy to prevent or mitigate chronic stress, and potentially burnout, among HCWs.

## Teaser

This randomized clinical trial assesses the efficacy of Transcendental Meditation practice over a 3-month period for the reduction of stress among health care workers.

## Introduction

Health care workers (HCWs) are experiencing substantial stress and burnout, with recently reported burnout rates of 60% to 70%.^1,2,3,4^ The World Health Organization defines burnout as an occupational phenomenon resulting from chronic workplace stress that has not been successfully managed.^5^ Burnout is characterized by 3 dimensions: feelings of exhaustion, increased mental distance from the job or negativism, and reduced professional efficacy.^6^ The serious consequences of burnout, such as rapid turnover, limited patient access and care, and increased health expenditure, can adversely affect HCWs, health care organizations, and patients.^3,6,7^ As a result, the Joint Commission and the Department of Health and Human Services have called for prioritization of health care workforce resilience.^8,9^ However, organizational burnout mitigation strategies have a limited evidence base with regard to approaches and strategies.

Several randomized clinical trials have evaluated strategies to reduce stress and burnout, such as the use of psychological or sensory-emotional techniques,^10,11,12,13^ group discussions,^14,15^ mindfulness- or compassion-based programs,^16,17,18,19^ auricular acupressure,^20^ and cannabidiol treatment.^21^ These studies reported some short-term improvements; however, small cohorts, high attrition rates, lack of sample diversity, or the need for pharmacological therapy has limited their utility, highlighting the need for rigorously tested interventions for burnout.

Transcendental Meditation (TM) is a meditation practice in which individuals silently recite a single mantra (a sound that lacks meaning) without concentration or contemplation.^22,23,24^ Studies of TM practitioners have revealed patterns of increased parasympathetic response leading to attenuation of the stress response.^22,23,25,26^ Proposed mechanisms underlying the restful alertness achieved with TM include increased α coherence on electroencephalography^27^ and increased blood flow to the prefrontal cortex on functional magnetic resonance imaging.^28^

Clinical trials have demonstrated the efficacy of TM for the reduction of stress and burnout among teachers^29^ and emergency department clinicians.^30^ The practice of TM has been found to reduce posttraumatic stress disorder symptoms in veterans,^31,32^ which might be particularly relevant because recent reports of HCW burnout suggest a similarity to the experiences of combat veterans.^33^

We hypothesized that HCWs who practiced TM would demonstrate significantly reduced symptoms of acute psychological distress within 3 months, as measured by the Global Severity Index (GSI) score of the 18-item Brief Symptom Inventory (BSI-18). Secondary outcomes included changes in burnout, resilience, insomnia, depression, and anxiety levels.

## Methods

### Study Design

This single-center open-label randomized clinical trial was conducted between November 19, 2020, and August 31, 2021, at Duke University Medical Center in Durham, North Carolina. The trial protocol and amendments (Supplement 1) were approved by the Duke University Health System Institutional Review Board. All participants provided written informed consent before participation. This study followed the Consolidated Standards of Reporting Trials (CONSORT) reporting guideline for randomized clinical trials.^34^

The goal was to evaluate the efficacy of TM compared with usual treatment for the reduction of stress and burnout among HCWs. The definition of HCWs was initially limited to physicians, physician trainees, advanced practice clinicians, and nurses; however, after randomization of 15 clinicians, the definition was expanded to include all patient-facing HCWs in response to reported worsening burnout among health care professionals at all levels.^35,36^ Participants were self-referred through flyers and author-led presentations on HCW burnout within the Duke Health System. Flyers were distributed in community facilities.

### Sample Size

This study was powered at 80% to detect an effect size of 0.6 in the GSI (the primary end point) at a significance level of α = .05. The effect size was based on the medium to large range of effect sizes of TM for psychological distress factors reported in previous studies and other interventions that used the GSI and other measures of stress.^29,37,38,39,40,41^

### Randomization

Participants were randomized using simple stratified randomization based on age (≥40 years vs <40 years) and sex (male vs female) consistent with reports of higher burnout and stress among female HCWs,^42^ higher risk of burnout among senior clinicians,^43^ and high acceptance of meditation practices among women and individuals older than 40 years.^44^ Participants were randomized on a 1:1 ratio to receive a 3-month TM intervention (TM group) or usual treatment comprising access to wellness resources (control group). A treatment allocation table was computer generated and uploaded to a password-protected randomization module in the Research Electronic Data Capture (REDCap) electronic case report form.

### Primary End Point

The primary outcome was the between-group (TM vs control) difference in the change in GSI scores (which represent total scores on the BSI-18) between baseline and 3 months. We selected the GSI as the primary outcome measure to assess acute stress because we hypothesized that TM would have the greatest impact on acute stress reduction, which in turn would decrease burnout.

### Secondary End Points

Secondary end points included between-group (TM vs control) differences in the change in scores on the Maslach Burnout Index (MBI) subscales (emotional exhaustion, depersonalization, and personal accomplishment),^45^ the Insomnia Severity Index (ISI),^46^ the 7-item Generalized Anxiety Disorder (GAD-7) scale,^47^ the 9-item Patient Health Questionnaire (PHQ-9),^48^ the BSI-18 subscales (somatization, depression, and anxiety),^49^ and the 10-item Connor-Davidson Resilience Scale (CD-RISC-10)^50,51^ from baseline to 3 months.

### Participant Population

Patient-facing HCWs were recruited from Duke University and the community. Interested participants viewed informative sessions on TM and completed prescreening for eligibility based on inclusion and exclusion criteria (eTable 1 in Supplement 2). Eligible participants attended an in-person screening (visit 1). After providing informed consent, participants completed the Columbia Suicide Severity Rating Scale (C-SSRS) and the 10-point Single Units of Distress Scale (SUDS); participants were excluded if results of the C-SSRS indicated suicidal ideation within the past 3 months or if they had a SUDS score lower than 6. The remaining participants were included if they had a 5% or greater increase in baseline heart rate or a 33% or greater increase in galvanic skin response after exposure to a personalized stressful script. This final cohort completed standardized rating scales and provided information on demographic characteristics and mental health history (Supplement 1) before randomization. Participants completed standardized scales online at 1 month (visit 2) and in person at 3 months (visit 3).

### Intervention

Participants from the TM arm received 5 days of instruction (Supplement 1). Session 1 comprised 1:1 training from a certified TM teacher. Sessions 2 to 5 were group sessions of 75 minutes each followed by self-practice of 20 minutes twice daily. There were 3 follow-up sessions with the TM teacher over the intervention period (a total of 8 sessions). Protocol fidelity was ensured by the delivery of instructions by a single TM teacher for most participants and by further supervision of the protocol by a senior TM teacher. The control group had access to wellness resources, such as mindfulness-based stress reduction, journaling workshops, stress and resilience training, and access to fitness and nutrition consultations, offered by the Duke University Health System.

### Measures

The BSI-18^49^ was used to assess psychological distress over the past 7 days. This inventory is designed primarily as a highly sensitive screen for psychiatric disorders and secondarily as an instrument to measure treatment outcomes. It evaluates symptoms based on responses to 18 items in 3 categories: somatization (6 items), depression (6 items), and anxiety (6 items). Each item response is assigned a value of 0 (not true at all) to 4 (true nearly all the time), with scores for each subscale ranging from 0 to 24 points. The total score, GSI, is calculated by adding the 3 subscale sums (range, 0-72 points) and summarizes the respondent’s overall level of psychological distress, with higher scores indicating higher distress (scores of ≥63 points are considered indicative of high distress level).

The MBI^45^ was used to measure participant burnout. This 22-item inventory measures emotional exhaustion (9 items), depersonalization (5 items), and personal accomplishment (8 items). Respondents indicate how often they experienced each statement on a 7-point scale ranging from never to every day. Higher scores indicate higher ratings of that factor. The criterion for burnout is the presence of emotional exhaustion subscores of 27 points or higher, depersonalization subscores of 13 points or higher, and personal accomplishment subscores of 33 points or lower. For emotional exhaustion, ratings are categorized as low (0-16 points), moderate (17-26 points), or high (≥27 points). For depersonalization, ratings are categorized as low (0-6 points), moderate (7-12 points), or high (≥13 points). For personal accomplishment, ratings are categorized as low (≤31 points), moderate (32-38 points), or high (≥39 points).

The GAD-7^47^ was used to measure anxiety symptoms. Participants rated the frequency of symptoms over the previous 2-week period from 0 (not at all) to 3 (nearly every day), with total scores indicating no symptoms (0-5 points), mild symptoms (6-10 points), moderate symptoms (11-15 points), or severe symptoms (≥15 points).

The PHQ-9^48^ was used to measure depression symptoms. Participants rated the frequency of symptoms over the previous 2-week period from 0 (not at all) to 3 (nearly every day), with total scores indicating no symptoms (0-5 points), mild symptoms (6-10 points), moderate symptoms (11-15 points), or severe symptoms (≥15 points).

The CD-RISC-10^50,51^ was used to measure psychological resilience. It contains 10 items rated on a 5-point Likert scale ranging from 0 (not true at all) to 4 (true nearly all the time). Possible scores range from 0 to 40 points, with higher scores indicating higher resilience.

The ISI^46^ was used to evaluate symptoms of insomnia. It is a 7-item questionnaire assessing sleep over the last month. A 5-point Likert scale is used to rate each item, with 0 indicating no problem and 4 indicating a very severe problem. The total score is interpreted as no insomnia (0-7 points), subthreshold insomnia (8-14 points), moderate insomnia (15-21 points), and severe insomnia (22-28 points).

Adherence to the TM intervention, as assessed by a TM teacher, was defined as (1) treatment session adherence, comprising attendance at at least 6 of 8 sessions (75%), and (2) TM home practice adherence, comprising meditation at least once per day on average.^30,31^

### Statistical Analysis

Statistical analyses were conducted using SAS software, version 9.4 (SAS Institute, Inc). The main analyses of the primary and secondary end points were based on a repeated-measures analysis framework using the SAS MIXED procedure. The analysis code is provided in eMethods in Supplement 2. The model for each end point included a common intercept for both treatment groups, indicator variables for month 1 and month 3, and interactions between these time variables and an indicator variable for the treatment group. Each model also included age and sex as covariates.

Model fit contrasts were used to estimate within-group and between-group differences at 1 month vs baseline and 3 months vs baseline. The model assumed an unstructured covariance structure for within-participant correlation, and we used the Kenward-Roger denominator degrees of freedom for the contrast *t* statistics.^40^ We assessed statistical significance using 2-tailed tests with α = .05 and reported 95% CIs corresponding to all estimates. We calculated means, SDs, medians, IQRs, and minimum and maximum values for all end points at baseline among all participants and at 3 months by treatment group. We calculated standardized effect sizes using the Carlson-Schmidt approach,^52^ which is appropriate for assessing between-group effect sizes using pretreatment vs posttreatment measurements (ie, baseline vs 3 months).

In post hoc analyses, we mapped participants’ GSI scores and BSI-18 subscale scores with normalized *t* scores based on community norms by sex.^49^ We described the distribution of the normalized *t* scores at baseline and assessed between-group differences in baseline vs 3-month scores using the repeated-measures framework with the same settings specified in the previous paragraph. Analyses of the 2 treatment groups were conducted per the intention-to-treat principle. No adjustments for multiple testing were made. Self-identified race and ethnicity data were collected based on previous reports of exacerbated stress and burnout among racial and ethnic minority individuals.^53,54^

## Results

### Participant Characteristics

Characteristics of participants randomized to the TM and control groups are shown in Table 1. Among 80 participants, 66 (82.5%) were women and 14 (17.5%) were men, with a mean (SD) age of 40 (11) years. One participant (1.3%) was American Indian or Alaska Native, 5 (6.3%) were Asian, 12 (15.0%) were Black, 59 (73.8%) were White, and 3 (3.8%) were of unknown or unreported race; 4 participants (5.0%) were Hispanic, and 76 (95.0%) were non-Hispanic. A total of 41 participants were randomized to the TM group, and 39 were randomized to the control group. Participants in the TM group vs the control group were similar in age (mean [SD], 39 [12] years vs 41 [9] years), sex (34 women [82.9%] vs 32 women [82.1%]), race (28 White individuals [68.3%] vs 31 White individuals [79.5%]), ethnicity (39 non-Hispanic individuals [95.1%[ vs 37 non-Hispanic individuals [94.9%]), and mental health history (eg, ever visited a psychiatrist or mental health worker: 28 individuals [68.3%] vs 28 individuals [71.8%]). The longitudinal adherence rate in the TM group was 92.7% (38 participants), with 27 participants (65.9%) practicing TM twice daily and 11 participants (26.8%) practicing TM at least once daily (eFigure in Supplement 2).

**Table 1.** Baseline Characteristics of Participants

Characteristic	Participants, No./total No. (%)		
	Total (N = 80)	TM group (n = 41)	Control group (n = 39)
Age, y			
Mean (SD)	40 (11)	39 (12)	41 (9)
Median (IQR) [range]	41 (31-46) [22-65]	39 (30-46) [22-65]	42 (36-46) [23-56]
Sex			
Female	66/80 (82.5)	34/41 (82.9)	32/39 (82.1)
Male	14/80 (17.5)	7/41 (17.1)	7/39 (17.9)
Self-identified race			
American Indian or Alaska Native	1/80 (1.3)	0	1/39 (2.6)
Asian	5/80 (6.3)	3/41 (7.3)	2/39 (5.1)
Black or African American	12/80 (15.0)	8/41 (19.5)	4/39 (10.3)
White	59/80 (73.8)	28/41 (68.3)	31/39 (79.5)
Unknown or not reported	3/80 (3.8)	2/41 (4.9)	1/39 (2.6)
Self-identified ethnicity			
Hispanic	4/80 (5.0)	2/41 (4.9)	2/39 (5.1)
Non-Hispanic	76/80 (95.0)	39/41 (95.1)	37/39 (94.9)
Marital status			
Single	31/80 (38.8)	19/41 (46.3)	12/39 (30.8)
Married	40/80 (50.0)	18/41 (43.9)	22/39 (56.4)
Partner	2/80 (2.5)	2/41 (4.9)	0
Separated	1/80 (1.3)	1/41 (2.4)	0
Divorced	5/80 (6.3)	1/41 (2.4)	4/39 (10.3)
Widowed	1/80 (1.3)	0	1/39 (2.6)
No. of members in household			
1	11/80 (13.8)	6/41 (14.6)	5/39 (12.8)
2	27/80 (33.8)	14/41 (34.1)	13/39 (33.3)
3	17/80 (21.3)	10/41 (24.4)	7/39 (17.9)
4	18/80 (22.5)	7/41 (17.1)	11/39 (28.2)
5	6/80 (7.5)	3/41 (7.3)	3/39 (7.7)
6	1/80 (1.3)	1/41 (2.4)	0
Smoking status			
Never	62/80 (77.5)	33/41 (80.5)	29/39 (74.4)
Past	16/80 (20.0)	8/41 (19.5)	8/39 (20.5)
Current	2/80 (2.5)	0	2/39 (5.1)
Current use of electronic cigarettes	2/80 (2.5)	0	2/39 (5.1)
Any current or past problem with alcohol	3/80 (3.8)	1/41 (2.4)	2/39 (5.1)
Current or past illicit or recreational drug use	8/80 (10.0)	5/41 (12.2)	3/39 (7.7)
Ever visited a psychiatrist or mental health worker			
No	24/80 (30.0)	13/41 (31.7)	11/39 (28.2)
Yes	56/80 (70.0)	28/41 (68.3)	28/39 (71.8)
Among those who ever visited a psychiatrist or mental health worker			
Condition			
Any anxiety	26/56 (46.4)	13/28 (46.4)	13/28 (46.4)
Any trauma or PTSD	2/56 (3.6)	0	2/28 (7.1)
Any depression	26/56 (46.4)	13/28 (46.4)	13/28 (46.4)
Any ADD or ADHD	4/56 (7.1)	3/28 (10.7)	1/28 (3.6)
Other conditions or none of the above	14/56 (25.0)	5/28 (17.9)	9/28 (32.1)
Diagnosis			
Anxiety	17/56 (30.4)	11/28 (39.3)	6/28 (21.4)
Trauma or PTSD	2/56 (3.6)	0	2/28 (7.1)
Depression	17/56 (30.4)	7/28 (25.0)	10/28 (35.7)
ADD or ADHD	4/56 (7.1)	3/28 (10.7)	1/28 (3.6)
Other conditions or none of the above	3/56 (5.4)	2/28 (7.1)	1/28 (3.6)
No diagnosis or unknown diagnosis	26/56 (46.4)	11/28 (39.3)	15/28 (53.6)
Treatment			
Medication	22/56 (39.3)	11/28 (39.3)	11/28 (39.3)
Therapy or counseling	18/56 (32.1)	7/28 (25.0)	11/28 (39.3)
Medication and therapy or counseling	7/56 (12.5)	4/28 (14.3)	3/28 (10.7)
Not treated	2/56 (3.6)	2/28 (7.1)	0
Resolved	17/56 (30.4)	8/28 (28.6)	9/28 (32.1)
Onset of condition			
2020-2021	5/56 (8.9)	2/28 (7.1)	3/28 (10.7)
Before 2020	51/56 (91.1)	26/28 (92.9)	25/28 (89.3)

Abbreviations: ADD, attention-deficit disorder; ADHD, attention-deficit/hyperactivity disorder; PTSD, posttraumatic stress disorder; TM, Transcendental Meditation.

### Recruitment

Of 279 participants who completed prescreening questionnaires, 66 were not eligible for participation based on inclusion and exclusion criteria (Figure; eTable 1 in Supplement 2). Among 213 eligible participants, 95 attended the in-person screening after providing informed consent. One participant was excluded due to the use of antipsychotic or β blocker medications, 2 were excluded due to current suicidal ideation (based on C-SSRS results), 7 scored less than 6 points on the SUDS, and 5 did not have either a 5% or greater increase in heart rate or a 33% or greater increase in galvanic skin response after exposure to a stressful script. Due to an error in the online survey app, a large number of participants (12 in the TM group and 20 in the control group) missed the time frame to complete the 1-month follow-up survey, leading to a high number of missing responses.

**Figure.**
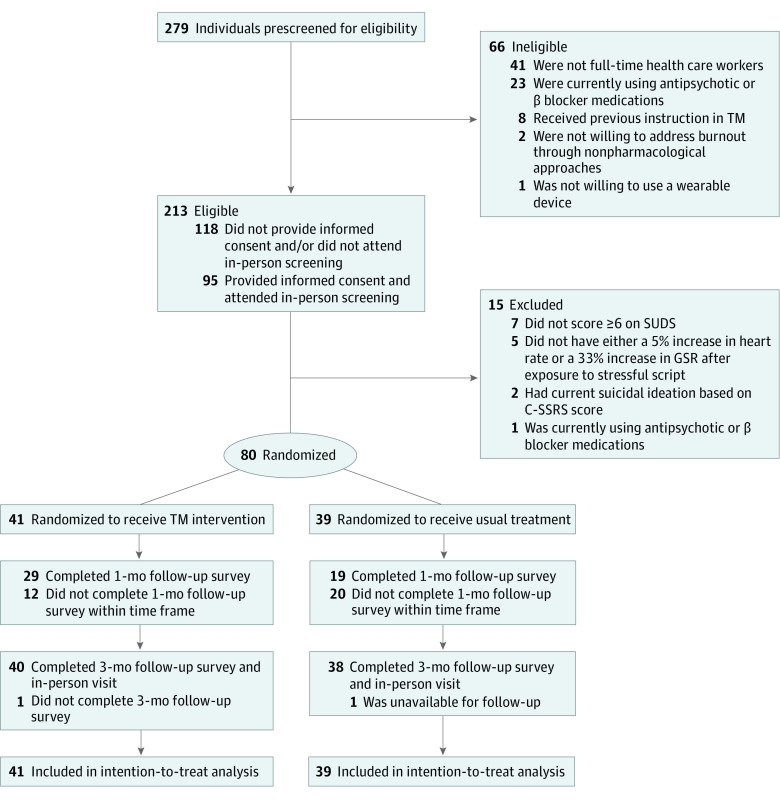
Study Flowchart C-SSRS indicates Columbia Suicide Severity Rating Scale; GSR, galvanic skin response; SUDS, Subjective Units of Distress Scale; and TM, Transcendental Meditation.

### End Point Measures at Baseline

Questionnaire outcomes for all participants at baseline and the TM and control groups are found in Table 2. At baseline, the cohort exhibited low levels of acute psychological distress (GSI score: median [IQR], 9 [4-15] points). However, participants had high levels of clinical burnout^55^ (MBI emotional exhaustion subscore: median [IQR], 29 [23-37] points), mild depression (PHQ-9 score: median [IQR], 7 [4-10] points), subthreshold insomnia (ISI score: median [IQR], 10 [7-14] points), and mild anxiety (GAD-7 score: median [IQR], 5 [3-10] points).

**Table 2.** Questionnaire Outcomes

Outcome	All participants at baseline (N = 80)	TM group at 3 mo (n = 40)	Control group at 3 mo (n = 38)
GSI score^a^			
Mean (SD)	10.3 (9.2)	4.1 (5.5)	7.3 (7.8)
Median (IQR) [range]	9 (4-15) [0-58]	3 (1-5) [0-26]	5 (0-11) [0-32]
BSI-18 somatization subscore^b^			
Mean (SD)	1.7 (2.4)	0.6 (0.9)	1.6 (2.3)
Median (IQR) [range]	1 (0-3) [0-14]	0 (0-1) [0-3]	1 (0-3) [0-10]
BSI-18 depression subscore^b^			
Mean (SD)	4.2 (4.4)	2.0 (3.4)	3.1 (3.7)
Median (IQR) [range]	3 (1-7) [0-22]	1 (0-3) [0-17]	2 (0-4) [0-15]
BSI-18 anxiety subscore^b^			
Mean (SD)	4.5 (4.0)	1.6 (2.1)	2.6 (3.0)
Median (IQR) [range]	4 (2-6) [0-22]	1 (0-3) [0-9]	2 (0-4) [0-13]
MBI emotional exhaustion subscore^c^			
Mean (SD)	29.0 (10.7)	20.2 (9.9)	26.9 (11.8)
Median (IQR) [range]	29 (23-37) [0-51]	22 (11-27) [6-50]	24 (18-37) [4-46]
MBI depersonalization subscore^c^			
Mean (SD)	7.5 (5.0)	4.6 (3.9)	6.5 (5.4)
Median (IQR) [range]	7 (3-10) [0-21]	4 (2-7) [0-13]	5 (4-9) [0-25]
MBI personal accomplishment subscore^c^			
Mean (SD)	35.0 (7.3)	39.7 (6.2)	36.2 (6.2)
Median (IQR) [range]	36 (30-41) [20-46]	41 (37-43) [20-48]	36 (31-42) [24-48]
ISI score^d^			
Mean (SD)	10.0 (4.9)	5.3 (4.8)	8.7 (6.6)
Median (IQR) [range]	10 (7-14) [0-21]	5 (2-9) [0-18]	7 (3-15) [0-22]
CD-RISC-10 score^e^			
Mean (SD)	27.1 (5.7)	30.9 (5.7)	28.5 (5.8)
Median (IQR) [range]	27 (24-31) [12-40]	30 (27-36) [21-40]	29 (24-31) [16-39]
PHQ-9 score^f^			
Mean (SD)	7.0 (4.5)	4.1 (4.4)	6.5 (5.3)
Median (IQR) [range]	7 (4-10) [0-21]	3 (1-6) [0-20]	5 (3-10) [0-19]
GAD-7 score^g^			
Mean (SD)	6.8 (5.2)	3.7 (3.9)	5.9 (5.0)
Median (IQR) [range]	5 (3-10) [0-21]	3 (1-4) [0-18]	5 (2-9) [0-19]

Abbreviation: TM, Transcendental Meditation.

^a^ Global Severity Index (GSI) scores range from 0 to 72 points, with higher scores indicating greater distress.

^b^ Brief Symptom Inventory-18 (BSI-18) subscale scores range from 0 to 24 points, with higher scores indicating greater distress.

^c^ Maslach Burnout Index (MBI) emotional exhaustion subscale scores range from 0 to 54 points (with higher scores indicating greater emotional exhaustion), depersonalization subscale scores range from 0 to 30 points (with higher scores indicating greater depersonalization), and personal accomplishment subscale scores range from 0 to 48 points (with higher scores indicating a greater sense of personal accomplishment).

^d^ Insomnia Severity Index (ISI) scores range from 0 to 28 points, with higher scores indicating more severe insomnia.

^e^ Connor-Davidson Resilience Scale-10 (CD-RISC-10) scores range from 0 to 40 points, with higher scores indicating higher resilience.

^f^ Patient Health Questionnaire-9 (PHQ-9) scores range from 0 to 27 points, with higher scores indicating more severe depression symptoms.

^g^ Generalized Anxiety Disorder-7 (GAD-7) scale scores range from 0 to 21 points, with higher scores indicating more severe anxiety symptoms.

### Follow-up at 3 Months

No adverse events were reported during the study period. The attrition rate at 3 months was 2 of 80 participants (2.5%), with 1 of 41 participants in the TM group unavailable for follow-up and 1 of 39 participants in the control group withdrawing from the study. Effect size estimates are shown in Table 3. The effect size of TM practice on reduction in GSI scores (primary end point) was small and not significant (−0.02; 95% CI, −0.47 to 0.43). The practice of TM had the largest effects on reducing MBI emotional exhaustion subscores (−0.49; 95% CI, −0.94 to −0.04) and ISI scores (−0.38; 95% CI, −0.83 to 0.07), although the effect size for ISI scores was not statistically significant.

**Table 3.** Effect Sizes for Primary and Secondary Outcomes

Outcome^a^	Effect size (95% CI)^b^
GSI^c^	−0.02 (−0.47 to 0.43)
BSI-18 subscales^c^	
Somatization	0.07 (−0.38 to 0.52)
Depression	0.07 (−0.38 to 0.52)
Anxiety	−0.16 (−0.61 to 0.29)
MBI subscales^c^	
Emotional exhaustion	−0.49 (−0.94 to −0.04)
Depersonalization	−0.32 (−0.77 to 0.13)
Personal accomplishment^d^	0.09 (−0.37 to 0.54)
ISI^c^	−0.38 (−0.83 to 0.07)
CD-RISC-10^d^	0.25 (−0.20 to 0.70)
PHQ-9^c^	−0.31 (−0.76 to 0.14)
GAD-7^c^	−0.35 (−0.80 to 0.10)

Abbreviations: BSI-18, Brief Symptom Inventory-18; CD-RISC-10, 10-item Connor-Davidson Resilience Scale; GAD-7, 7-item Generalized Anxiety Disorder scale; GSI, Global Severity Index; ISI, Insomnia Severity Index; MBI, Maslach Burnout Index; PHQ-9, 9-item Patient Health Questionnaire.

^a^ The primary outcome was GSI score. All other scores were secondary outcomes.

^b^ Effect size estimates were calculated using the Carlson-Schmidt method^52^ for paired pretreatment and posttreatment data. Effect sizes were calculated based on changes in outcomes between baseline and 3 months. A negative effect size indicates lower scores in the Transcendental Meditation (TM) group than the control group at 3 months compared with baseline. A positive score indicates higher scores in the TM group than the control group at 3 months compared with baseline.

^c^ Negative effect sizes in these measures indicate better outcomes in the TM group than the control group.

^d^ Positive effect sizes in these measures indicate better outcomes in the TM group than the control group.

Estimates of mean changes between baseline and 3 months obtained from the repeated-measures analysis are reported in Table 4. Participants in the TM group showed a greater decrease in psychological distress (primary outcome measure based on GSI score) than participants in the control group, but the between-group difference was not statistically significant (−5.6 points vs −3.8 points; between-group difference, −1.8 points; 95% CI, −4.2 to 0.6 points; *P* = .13). Participants in the TM group vs the control group had significantly larger decreases in MBI emotional exhaustion subscores (−8.0 points vs −2.6 points; between-group difference, −5.4 points; 95% CI, −9.2 to −1.6 points; *P* = .006), ISI scores (−4.1 points vs −1.9 points; between-group difference, −2.2 points; 95% CI, −4.4 to 0 points; *P* = .05), and GAD-7 scores (−3.1 points vs −0.9 points; between-group difference, −2.2 points; 95% CI, −3.8 to −0.5 points; *P* = .01). Compared with participants in the control group, those in the TM group had larger but nonsignificant decreases in MBI depersonalization subscores (−2.8 points vs −1.1 points; between-group difference, –1.7 points; 95% CI, −3.6 to 0.2 points; *P* = .08) and PHQ-9 scores (−2.7 points vs −0.9 points; between-group difference, −1.8 points; 95% CI, −3.7 to 0.1 points; *P* = .06). Participants in the TM group also had larger but nonsignificant increases in CD-RISC-10 scores (3.3 points vs 1.8 points; between-group difference, 1.5 points; 95% CI, −0.6 to 3.7 points; *P* = .16) and MBI personal accomplishment subscores (3.9 points vs 2.1 points; between-group difference, 1.9 points; 95% CI, −0.4 to 4.1 points; *P* = .11) compared with those in the control group.

**Table 4.** Estimated Mean Changes Between Baseline and 3 Months

Outcome^a^	Estimated change between baseline and 3 mo, mean (95% CI)^b^			
	TM group	Control group	TM vs control group	*P* value for TM vs control group^**c**^
GSI^d^^,^^e^	−5.6 (−7.5 to −3.6)	−3.8 (−5.7 to −1.8)	−1.8 (−4.2 to 0.6)	.13
BSI-18 subscales^e^^,^^f^				
Somatization	−0.9 (−1.4 to −0.4)	−0.4 (−0.9 to 0.1)	−0.5 (−1.1 to 0.1)	.13
Depression	−1.9 (−3.0 to −0.8)	−1.4 (−2.5 to −0.3)	−0.6 (−1.9 to 0.8)	.42
Anxiety	−2.8 (−3.7 to −1.9)	−1.9 (−2.8 to −1.0)	−0.9 (−1.8 to 0.1)	.06
MBI subscales^e^^,^^g^				
Emotional exhaustion	−8.0 (−10.7 to −5.3)	−2.6 (−5.4 to 0.2)	−5.4 (−9.2 to −1.6)	.006
Depersonalization	−2.8 (−4.2 to −1.3)	−1.1 (−2.6 to 0.4)	−1.7 (−3.6 to 0.2)	.08
Personal accomplishment^h^	3.9 (2.2 to 5.7)	2.1 (0.3 to 3.9)	1.9 (−0.4 to 4.1)	.11
ISI^e^^,^^i^	−4.1 (−5.6 to −2.5)	−1.9 (−3.5 to −0.3)	−2.2 (−4.4 to 0)	.05
CD-RISC-10^h^^,^^j^	3.3 (1.7 to 4.9)	1.8 (0.2 to 3.4)	1.5 (−0.6 to 3.7)	.16
PHQ-9^e^^,^^k^	−2.7 (−4.1 to −1.3)	−0.9 (−2.3 to 0.5)	−1.8 (−3.7 to 0.1)	.06
GAD-7^e^^,^^l^	−3.1 (−4.3 to −1.8)	−0.9 (−2.2 to 0.3)	−2.2 (−3.8 to −0.5)	.01

^a^ The primary outcome was Global Severity Index (GSI) score. All other scores were secondary outcomes.

^b^ Estimates were obtained using a repeated-measures modeling framework (complete model specifications are available in eMethods in Supplement 2).

^c^ The significance threshold was *P* = .05.

^d^ GSI scores range from 0 to 72 points, with higher scores indicating greater distress.

^e^ Negative estimates in these measures indicate improving outcomes between baseline and 3 months and greater improvements in the Transcendental Meditation (TM) group compared with the control group.

^f^ Brief Symptom Inventory-18 (BSI-18) subscale scores range from 0 to 24 points, with higher scores indicating greater distress.

^g^ Maslach Burnout Index (MBI) emotional exhaustion subscale scores range from 0 to 54 points (with higher scores indicating greater emotional exhaustion), depersonalization subscale scores range from 0 to 30 points (with higher scores indicating greater depersonalization), and personal accomplishment subscale scores range from 0 to 48 points (with higher scores indicating a greater sense of personal accomplishment).

^h^ Positive estimates in these measures indicate improving outcomes between baseline and 3 months and greater improvements in the TM group compared with the control group.

^i^ Insomnia Severity Index (ISI) scores range from 0 to 28 points, with higher scores indicating more severe insomnia.

^j^ Connor-Davidson Resilience Scale-10 (CD-RISC-10) scores range from 0 to 40 points, with higher scores indicating higher resilience.

^k^ Patient Health Questionnaire-9 (PHQ-9) scores range from 0 to 27 points, with higher scores indicating more severe depression symptoms.

^l^ Generalized Anxiety Disorder-7 (GAD-7) scale scores range from 0 to 21 points, with higher scores indicating more severe anxiety symptoms.

### Secondary Analyses

In the repeated-measures analysis of changes between baseline and 1 month (eTable 2 in Supplement 2), we observed a statistically significant between-group difference in PHQ-9 scores (−2.2 points; 95% CI, −3.9 to −0.4 points; *P* = .02) and GAD-7 scores (−2.1 points; 95% CI, −3.8 to −0.3 points; *P* = .02) at 1 month. In the analysis of normalized GSI scores and BSI-18 subscores, few participants (3 in the TM group and 6 in the control group) reached the threshold for a high distress level (≥63 points on the GSI) at baseline (eTable 3 in Supplement 2). The repeated-measures analysis of normalized scores showed results similar to the analysis of raw scores (eg, between-group difference in normalized GSI scores: −2.0 points; 95% CI, −5.6 to 1.6 points; *P* = .27) (eTable 4 in Supplement 2)

## Discussion

This randomized clinical trial did not find a significant reduction in the primary outcome of acute psychological distress among HCWs practicing TM over a 3-month period compared with those receiving usual treatment. However, participants in the TM group experienced significant reductions in chronic stress, particularly emotional exhaustion of burnout (a secondary end point), compared with those in the control group. Our findings on burnout are supported by a recent study reporting decreases in burnout among emergency medicine clinicians practicing TM^30^ as well as studies reporting reductions in burnout in other vulnerable professions, such as teaching and nursing.^29,41^ Our findings were comparable with those reported in studies of other interventions, such as coaching^56^ or psychological techniques to improve focus on positive emotions,^13^ but our study had lower longitudinal attrition (2.5%), higher adherence (92.7% in the TM group), and a notable lack of adverse events compared with those studies. We found significant decreases in insomnia, anxiety, and emotional exhaustion levels (secondary end points) in the TM group compared with the control group. However, the improvement patterns in depression symptoms, depersonalization, and personal accomplishment between the TM group vs the control group did not reach statistical significance.

There could be several reasons for the absence of significant improvement in our primary end point of acute psychological distress despite the observed reduction in burnout. This finding may represent a floor effect (which occurs when a large percentage of respondents score near the lower limit on a questionnaire). At baseline, the cohort had low GSI scores (median [IQR], 9 [4-15] points) and thus may not have been able to further reduce symptoms in a 3-month period. Only 3 participants in the TM group and 6 participants in the control group had a high standard GSI score (≥63 points) at baseline,^49^ and these small numbers were inadequate for statistical analysis. Given that the cohort had high levels of burnout at baseline but low acute distress scores, prioritized strategies for mitigation of chronic stress rather than acute distress may be needed for HCWs. It is also possible that the use of a different psychological instrument may be warranted to evaluate acute stress among HCWs. Although the GSI was developed as a highly sensitive screening measure for acute psychological distress and as an outcome measure for change in stress levels among individuals recovering from cancer,^49^ it is not reliable in practice for the longitudinal identification of psychological distress across populations.^57,58^ Thus, the GSI may be an inadequate instrument for measuring stress levels among HCWs. Our results showed a significant effect of TM practice on the MBI emotional exhaustion subscale, which has been reported by other studies that used the MBI.^55,59^

Our study had a higher number of female participants, which is consistent with recent reports of disproportionately high burnout in the female workforce^36,60^ and greater willingness to take action against burnout among women.^6^ This finding highlights the need to address underlying factors and develop organizational mitigation strategies. The cost of interventions such as TM could be prohibitive for individual HCWs. Thus, organizations and payers may consider offering processes and subsidies to HCWs who choose to address their burnout with neurobehavioral tools. Future studies might recruit more participants to ensure they have the necessary power to detect significant changes in numerical scales, and they could measure serum markers of stress, such as inflammatory cytokines and cortisol levels.

### Strengths and Limitations

This study has several strengths. To our knowledge, this randomized clinical trial is one of the largest to assess the practice of TM for stress reduction among HCWs. We used validated scales and objective measures of distress, such as galvanic skin response and increased heart rate. We recruited a variety of HCWs (not only physicians), giving the findings’ greater generalizability, and conducted 3 months of longitudinal follow-up with low attrition rates.

This study also has limitations. The study cohort was small and possibly comprised a highly motivated group, and the study lacked long-term assessments beyond 3 months. In a larger cohort, TM practice may yield statistically significant differences from usual treatment, even for acute psychologic distress. The control group received access to wellness resources rather than an active intervention. Our study was also limited in terms of knowledge of objective intervention adherence and quality. The initial 5-day TM instruction was relatively intense, and abbreviated training or flexible schedules may enhance participation among HCWs.

## Conclusions

This randomized clinical trial found that TM practice may not significantly reduce acute psychological distress among HCWs compared with usual treatment. However, TM may significantly alleviate burnout, anxiety, and insomnia among HCWs. The findings also revealed high stress levels among HCWs, especially women, who compose more than 50% of the health care workforce. It is important that investigators and organizations use interdisciplinary multidimensional approaches that incorporate personal and organizationally led strategies. The practice of TM, which is both feasible and safe, could be considered as 1 strategy to prevent or mitigate chronic stress and burnout.

## Notes

Supplement 1.
Trial Protocol and Statistical Analysis Plan

Supplement 2. **eMethods.** Supplemental Code **eTable 1.** Inclusion and Exclusion Criteria **eTable 2.** Estimated Mean Changes Between Baseline and 1 Month in Transcendental Meditation and Control Groups **eTable 3.** Normalized GSI Score and BSI-18 Subscores at Baseline **eTable 4.** Estimated Mean Changes in Normalized GSI and BSI Subscores Between Baseline and 1 Month in Transcendental Meditation and Control Groups **eFigure.** Summary of Adherence Among Participants in the Transcendental Meditation Group

Supplement 3.
Data Sharing Statement
