# Supplementary material for: Efficacy of Transcendental Meditation to Reduce Stress Among Health Care Workers: A Randomized Clinical Trial
Source: JAMA Netw Open. 2022 Sep 19;5(9):e2231917. doi: 10.1001/jamanetworkopen.2022.31917 (PMC9486450; doi:10.1001/jamanetworkopen.2022.31917)
Supplement: Supplement 1. — Trial Protocol and Statistical Analysis Plan [file jamanetwopen-e2231917-s001.pdf]

DUHS IRB Application (Version 1.21)

General Information

***Please enter the full title of your protocol:**

Targeting the Health Care Provider (HCP) Burnout Crisis during the COVID Pandemic

***Please enter the Short Title you would like to use to reference the study:**

Marcus Foundation
* This field allows you to enter an abbreviated version of the Study Title to quickly identify this study.

Add Study Organization(s):

List Study Organizations associated with this protocol:

Primary Dept?	Department Name
○	**DUHS** - Medicine-Pulmonary

Assign key study personnel (KSP) access to the protocol

*** Please add a Principal Investigator for the study:**

(Note: Before this study application can be submitted, the PI MUST have completed CITI training)

Lee, Patty

3.1 **If applicable, please select the Key Study personnel: (Note: Before this study application can be submitted, all Key Personnel MUST have completed CITI training)**
*** Denotes roles that are not recognized in OnCore. Please select an appropriate role that is recognized in all clinical research applications (iRIS, OnCore, eREG, etc.)**

A) Additional Investigators, Primary Study Coordinator (CRC), and the Primary Regulatory Coordinator (PRC):

Ardito, Taylor
Primary Study Coordinator (CRC/CRNC/RPL)
Foss, Catherine
Primary Study Coordinator (CRC/CRNC/RPL)
Joshi, Sangeeta
Co-PI
Kuehn, Heather
Primary Regulatory Coordinator

B) All Other Key Personnel

Brucker, Amanda
Statistician
Chow, Shein-Chung
Sub-Investigator
Dunn, Jessilyn
Analyst*
Harshbarger, Todd
Other*
Ratcliffe, Erika
Other*
Song, Allen
Other*
Wang, Ke
Graduate Student

***Please add a Study Contact:**

Ardito, Taylor
Foss, Catherine
Joshi, Sangeeta
Kuehn, Heather
Lee, Patty
Ratcliffe, Erika
Shier, Jessica

The Study Contact(s) will receive all important system notifications along with the Principal Investigator. (e.g., The study contact(s) are typically the Principal Investigator, Study Coordinator, and Regulatory Coordinator.)

Oncore

Please select the Library for your Protocol:

This field is used in OnCore. Determines the Reference Lists, Forms, Protocol Annotations, Notifications, and Signoffs available for the protocol. Protocols that require reporting to the NCI (National Cancer Institute), must select the Oncology library.

- ○ Oncology
- ◉ Non-Oncology

Protocol Application Type

Select the type of protocol you are creating:

Please see additional criteria and information in the policy titled "Reliance on the IRB of Another Institution, Organization, or an Independent IRB" on the **IRB web site**.

- ◉ Regular Study Application - Most common. The IRB will determine if the study is eligible for expedited review or requires full board review upon submission.
- ○ Application for Exemption from IRB Review - Includes Exempt, Not Human Subject Research, & Not Research.
- ○ External IRB Application - Any study using an external IRB as the IRB-of-Record.
- ○ Trainee Research While Away from Duke - Research conducted by medical students overseen by the Office of Curriculum & other student/trainee research away from Duke.
- ○ Individual Patient Expanded Access, Including Emergency Use - Use of an investigational product [illegible]

use of an unapproved device.

Conflict of Interest

Do any of the participating study investigators or other key personnel (or their immediate family/significant other) have a financial or intellectual interest in, or are receiving compensation from, the sponsor or the drugs, devices or technologies used in this research?

○ Yes ◉ No

Are any key personnel an inventor of any of the drugs, devices or technologies used in this research?

○ Yes ◉ No

Do any key personnel have or anticipate (within the year) any financial relationships (e.g., consulting, speaking, advisory boards, patents, equity, options) that could be perceived to overlap or present a conflict of interest with the current research?

○ Yes ◉ No

Do any key personnel have a conflict of interest management plan or cautionary memo issued by DOSI-COI related to this research?

○ Yes ◉ No

Oversight Organization Selection

CRU (Clinical Research Unit) or Oversight Organization Selection:

Please select the CRU.

Medicine

The Clinical Research Unit that takes responsibility for this study.

- Please select **Medicine** as the CRU **only** if the PI is in one of these Divisions or Institutes: Endocrinology, Gastroenterology, General Internal Medicine, Geriatrics, Hematology, Infectious Diseases, Nephrology, Pulmonary, Rheumatology & Immunology, Center for Applied Genomics and Precision Medicine, Center for the Study of Aging and Human Development, Duke Molecular Physiology Institute.
- More information on CRUs can be found on the Duke Office of Clinical Research (DOCR) website, **http://docr.som.duke.edu**
- Questions concerning CRU selection should be directed to docr.help@dm.duke.edu.
- For questions about the Campus Oversight Organization, please visit **Campus Oversight Organization**.

List all Key Personnel on the study who are outside Duke:

- **Note:** You will also need to attach the documentation of Human Subjects Certification for each individual, if they have completed the certification somewhere other than Duke.
- **If outside key personnel will have access to Duke PHI, a data transfer agreement AND external site IRB approval (or IRB authorization agreement) will be needed.** See HRPP policy **Use of Research Data by Former Duke Students or Former Duke Faculty and Employees**

- In the panel below, "PHI" is Protected Health Information.

Entry 1

**Name**	Sandeep Vaishnavi
**Study Role**	contracted neuropsychologist to administer questionnaires
**Email Address**	sandeep.vaishnavi@duke.edu
**Institution / Organization**	Independent Contractor
**Will he/she have access to Duke P.H.I.?**	(•) Yes ( ) No
**Is he/she an unpaid volunteer at Duke on the study?**	( ) Yes (•) No

Indicate the Protocol source below:

The protocol source is the author of the protocol. If the protocol is a joint authorship between multiple sources, select the primary author.

An IRB fee may be assessed for all research that is supported by for-profit entities and requires full board review. For additional information, see the **IRB fees section of the IRB web site**

(•) PI initiated
( ) Commercial / Industry (for-profit entity) initiated
( ) Federal Government initiated
( ) Cooperative Group Initiated
( ) Foundation (non-profit group) initiated
( ) Other

Sponsor and Funding Source

Add all funding sources for this study:

View Details	Sponsor Name	Sponsor Type	Contract Type:	Project Number	Award Number
⊟	Duke University	Institutional			

Sponsor Name:	Duke University
Sponsor Type:	Institutional
Sponsor Role:	
**Project Period:**	From: to:
**Is Institution the Primary Grant Holder:**	No
**if No, then who is the Primary Grantee?**	
Contract Type:	
Project Number:	
Award Number:	

PI Name:<br>(If PI is not the same as identified on the study.)	
Explain Any Significant Discrepancy:	

⊟	Marcus Foundation	Institutional			

Sponsor Name:	Marcus Foundation
Sponsor Type:	Institutional
Sponsor Role:	Funding
**Grant/Contract Number:**	
**Project Period:**	From: to:
**Is Institution the Primary Grant Holder:**	No
**if No, then who is the Primary Grantee?**	
Contract Type:	
Project Number:	
Award Number:	
Grant Title:	
PI Name:<br>(If PI is not the same as identified on the study.)	
Explain Any Significant Discrepancy:	

Is this a federally funded study?

○ Yes ◉ No

Does this study have any of the following?

- Industry sponsored protocol
- Industry funded Duke protocol
- Industry funded sub-contract from another institution
- Industry provided drug/device/biologic
- SBIR/STTR funded protocol

○ Yes ◉ No

As part of this study, will any samples or PHI be transferred to/from Duke to/from anyone other than the Sponsor, a Sponsor subcontractor, or a Funding Source?

○ Yes ◉ No

Is the Department of Defense (DOD) a funding source?

○ Yes ◉ No

of this page?

Please verify that the protocol has been created in OnCore before submitting this application for PI Signoff.

- (●) Yes, I synced my protocol to OnCore and verified it was successfully sent by logging into OnCore.
- ( ) I may have forgotten! I'll click it again right now, just to be sure, and verify it was successfully sent by logging into OnCore.

Mobile Devices and Software

Does this study involve the use of a software or a mobile application?

(●) Yes ( ) No

Please describe the following:

- The developer of the mobile app and how the app will be obtained.
- What PHI will be collected via the app.
- Where the data will be stored and who will have access to it.

1. The developer of the mobile app and how the app will be obtained.

   Apple Watches will be given to participants.
   Pattern Health will develop an iPhone app the will be accessed on the Apple Watch. The features:

- Capture Survey information:
  - Ability to deliver assessments, scores, and summarize the results. Deliver quizzes and/or patient reported outcome surveys based on a schedule
- Collect Physiologic information
  - Heart Rate Variability (HRV): Ability to collect HRV from the Apple Watch through Apple
  - HealthKit and make that data available through the Pattern Health Console and API's
  - Sleep
  - Steps
- A module will be created in the Pattern Health Platform for Transcendental Meditation/Experience Resolution Methodology with the following attributes:
  - Reminders to start TM by native app notifications, email, or SMS
  - Includes a timer that the user can start and stop to record the length of the TM session
  - End of TM Survey

The Pattern Health app is an iPhone app. It will be downloaded from the apple app store.

Where the data will be stored and who will have access to it.

- Data Privacy & Security: Data will be stored on the Pattern Health platform.

  The platform is HITRUST certified and provides a secure platform for both the providers and end-users.

- Authentication: Use of Pattern Health's basic authentication or integrate with Single Sign On (SSO)

List all software, including third party (non-Duke) and mobile apps, that will be utilized for ascertainment, recruitment, or conduct of the research/project: (eg, MaestroCare, DEDUCE):

- DUHS Server
- SAS v9.4 Analysis Files
- Duke Box: multi-factor authentication is currently used

- Duke Medicine WebEx. Duke OIT is the service owner for WebEx a third party service and no data is being recorded with its use (per Duke policy)
- Duke Medicine ZOOM. Duke OIT is the service owner for Zoom a third party service and no data is being recorded with its use (per Duke policy)
- REDCap
- Pattern Health contact for data transmission contact
  - Ed Barber ebarber@pattern.health
- Duke contact for data transmission
  - Shein-Chung Chow **sheinchung.chow@duke.edu**
- Facebook

Multi-site Research

Is this a multi-site study?

(•) Yes ( ) No

Is the Duke PI/Co-PI the lead investigator or primary grant awardee?

(•) Yes ( ) No

Is the primary grant awardee a Duke employee?

( ) Yes (•) No

Is a Duke employee the holder of the IND or IDE?

( ) Yes
( ) No
(•) N/A

Is Duke the central coordinating center for this study?

(•) Yes ( ) No

Is Duke serving as a central statistical center for this study?

( ) Yes (•) No

Is Duke serving as a central laboratory, reading center, analysis center or other central resource for this study?

( ) Yes (•) No

Do you have ten or more sites?

( ) Yes (•) No

Complete for each site if Duke is the Primary grant awardee or coordinating center:

Entry 1

**Site Name:**	Emory University
**City:**	Atlanta
**State/Province:**	GA
**Country:**	USA
	**Site Contact Information**
**Primary Contact Name:**	Jenny Han
**Primary Contact Phone:**	
**Primary Contact Email:**	jehan2@emory.edu
	**Site Details**
**Does the site have an IRB?**	(•) Yes ( ) No
**Site IRB approval expiration date:**	
**If date not provided, explanation of why:**	According to the Emory IRB communication on the approval document:<br>On 3/15/2021 the Emory IRB reviewed the above-referenced study by expedited process. This<br>research is eligible for expedited review under 45 CFR.46.110 and/or 21 CFR 56.110 because it<br>poses minimal risk and fits expedited review category F7 as set forth in the Federal Register.<br>No annual IRB review is required, as permitted under the 2018 Common Rule.
**Has the site granted permission for the research to be conducted?**	(•) Yes ( ) No
**Does the site plan to rely on the DUHS IRB for review?**	( ) Yes (•) No
**What is the status of the study at this site?**	(•) Open<br>( ) Closed
**Site approval letters or site personnel lists:**	Attach site approval letters, site closure letterS (if applicable), or site personnel lists in the Initial Submission Packet.

Provide a description of the procedures that will be used to inform sites of unanticipated problems involving risks to subjects or others, interim results, protocol modifications and other information that may be relevant to the protection of subjects:

If there are any unanticipated problems involving risks to subjects, interim results, protocol modifications or any other information that may be relevant to the protection of subjects, the Duke PI will document this information in a formal written communications to the site.

How will you ensure that management, data analysis, and data safety and monitoring systems are adequate, given the nature of the research involved?

Prior to site initiation, the site PI and staff will participate in an educational teleconference with coordinating site team to ensure that all personnel fully understand the protocol, data collection instruments, and any other study related issues or documents. Contact information, including PI and study coordinators phone numbers will be provided to the site.

How will you ensure that sample protocols and informed consent documents are developed and distributed to each collaborating institution?

The study protocol will be distributed to the site prior to IRB submission. The site will draft their ICF according to their institutions IRB requirements.

How will you ensure that each collaborating institution holds an applicable OHRP-approved Assurance?

The OHRP Database for Registered IORGs and IRBs

How will you ensure that each protocol is reviewed and approved by the IRB at the collaborating institution prior to the enrollment of subjects?

Duke will receive a copy of the institutions IRB approval letter and a copy of the approved ICF.

How will you ensure that any substantive modification by the collaborating institution of sample consent information related to risks or alternative procedures is appropriately justified?

This is a minimal risk study

How will you ensure that informed consent is obtained from each subject in compliance with DHHS regulations?

Prior to site initiation, the site PI and study staff will participate in an educational teleconference with Duke study team to ensure that all personnel fully understand the protocol data collection, and any other study related issues or documents. Duke study team contact information will be supplied to the site.

Research Abstract

Please type your Research Abstract here:

The Research Abstract should summarize the main points of your study in one paragraph. The following guidelines may help you:

1. Purpose and objective (1-2 sentences)
2. Study activities and population group (2-4 sentences)
3. Data analysis and risk/safety issues (1-2 sentences)

This is a single blind pilot Randomized Clinical Trial (RCT) of TM (Transcendental Meditation) vs an active control of Treatment as Usual (TAU) in health care workers managing COVID-19 patients.

The primary goal of this study is to evaluate the effectiveness of TM vs TAU to improve burnout symptoms in providers. The overall hypothesis is that HCPs who learn and practice TM will demonstrate significantly improved

symptoms of burnout within 3 months, as measured by self-reporting (survey), physiologic, and neuro-functional imaging studies.

This is a single-blind, randomized, controlled trial (RCT) of TM (N=64) vs. Control (CTL], N=64) in frontline HCPs. HCPs will be screened by a single-item stress scale and Columbia Suicide Severity Rating Scale (CSSRS). The Global Severity Index of the Brief Symptom Inventory (BSI)-18 Global Severity score will be used as the primary outcome for pre- and post-TM training (baseline, 1 vs. 3 months).

For all primary and secondary outcomes, data will be analyzed using the method of analysis of covariance (ANCOVA) with repeated measures at each battery of measurements for psychological distress, burnout, anxiety, depression, insomnia, resilience and blood rate variability-related heart diseases. Dependent variables will be changed from baseline at three-month and at six-month post-test with treatment group as the independent variable and baseline characteristics as covariates. Effect sizes will be calculated using Cohen's d: mean differences divided by the pooled standard deviation. In addition, the responder's analysis will be performed. A participant is defined as a responder if the participant meets a pre-specified criteria (50% improvement or greater) of improvement post-treatment at 1 month or at 3 months. Exploratory analyses such as subgroup analyses may be performed as deemed appropriate by the principal investigators or biostatisticians. For the primary outcome, change in total burnout, the significance level will be set at $p<0.05$, two-tailed.

Research Summary

State your primary study objectives

SPECIFIC AIM 1: To conduct a single-blind, randomized, controlled trial (RCT) of TM,Transcendental Meditation, (N=64) vs. Control (CTL, N=64) in frontline HCPs. HCPs will be screened by a single-item stress scale and Columbia Suicide Severity Rating Scale (CSSRS). The Global Severity Index of the Brief Symptom Inventory (BSI)-18 Global Severity score will be used as the primary outcome for pre- and postTM training (baseline, 1 vs. 3 months).

SPECIFIC AIM 2: Evaluate changes (pre/post-treatment) in heart rate variability (HRV), a physiological measure of stress, and overall cardiovascular resiliency, in response to TM practice versus CTL. Participants will be asked to wear an Apple Watch or Empatica device throughout trial participation to assess changes in resting HRV during meditative practice. Galvanic Skin Response (GSR) refers to the changes in sweat gland activity that result from changes in an emotional state. A portable GSR device (eSense Skin Response) will be used to evaluate GSR score (baseline and 3 months). *fMRI* (N=32 for each group) will be performed by the Duke Brain Imaging and Analysis Center (BIAC).

SPECIFIC AIM 3: Conduct a feasibility study of the proposed mobile app (Pattern Health Technologies, Inc) and Apple Watch or Empatica device. 10 healthy volunteers after consent, will utilize app and questionnaires as well as the apple watch for heart rate variability. Formal written feedback will be obtained on user friendliness as well as gap in functionality. This feedback will be utilized for refining and optimization of app development.

State your secondary study objectives

Please select your research summary form:

Standard Research Summary Template

This is the regular (generic) research summary template which is required for all regular applications (unless your protocol fits under the other research summary templates in this category). Use of these instructions is helpful for ensuring that the research summary contains all necessary elements.

Purpose of the Study

Objectives & hypotheses to be tested

Hypothesis: HCPs who learn and practice TM (Transcendental Meditation) will demonstrate significantly improved symptoms of burnout within 3 months, as measured by self-reporting (survey), physiologic, and neuro-functional imaging studies.

SPECIFIC AIM 1: To conduct a single-blind, randomized, controlled trial (RCT) of TM (N=64) vs. Control (CTL, N=64) in frontline HCPs. HCPs will be screened by a single-item stress scale and Columbia Suicide Severity Rating Scale (CSSRS). The Global Severity Index of the Brief Symptom Inventory (BSI)-18 Global Severity score will be used as the primary outcome for pre- and post-TM training (baseline, 1 vs. 3 months).

SPECIFIC AIM 2: Evaluate changes (pre/post-treatment) in heart rate variability (HRV), a physiological measure of stress, and overall cardiovascular resiliency, in response to TM practice versus CTL. Participants will be asked to wear an Apple Watch or or Empatica device throughout trial participation to assess changes in resting HRV during meditative practice. Galvanic Skin Response (GSR) refers to the changes in sweat gland activity that result from changes in an emotional state. A portable GSR device (eSense Skin Response) will be used to evaluate GSR score (baseline and 3 months). *fMRI* (N=32 for each group) will be performed by the Duke Brain Imaging and Analysis Center (BIAC).

SPECIFIC AIM 3: Conduct a feasibility study of the proposed mobile app (Pattern Health Technologies, Inc) and Apple Watch or Empatica device. Up to 10 healthy volunteers after consent, will utilize app and questionnaires as well as the apple watch or or Empatica device for heart rate variability. Formal written feedback will be obtained on user friendliness as well as gap in functionality. This feedback will be utilized for refining and optimization of app development.

Background & Significance

- Should support the scientific aims of the research

Healthcare providers (HSP) are battling dual pandemics, COVID-19 and burnout syndrome. The World Health Organization defines burnout as a “syndrome conceptualized as resulting from chronic workplace stress that has not been successfully managed”. Symptoms include exhaustion, negative feelings and distancing from a job, and reduced professional productivity. HCP burnout is a major concern in the United States; in 2019, 42% of physicians and almost 20% of nurses reported elevated symptoms of burnout. National organizations such as the Joint Commission have documented concerns about the provider and patient safety affected by burnout, have issued safety advisories against burnout, and have provided guidelines and strategies to improve risk, resilience, and recovery strategies. The COVID-19 pandemic has enhanced high baseline rates of HCP burnout rates in those treating critically ill patients. In addition, frontline HCPs are reporting increased symptoms of depression, anxiety, posttraumatic stress disorder (PTSD) and there have been several reported HCP suicides. This “second victim phenomenon” in HCPs may be further worsened by a second surge in COVID-19 by Fall 2020. This creates an urgent unmet need for a quick and effective intervention to address burnout in frontline HCPs.

One method that has shown promise to improve HCP burnout is Transcendental Meditation (TM), a mindbody intervention designed to reduce sympathetic arousal and promote a state of relaxation and calm. TM was recently demonstrated to be as effective as prolonged exposure for the treatment of PTSD and has been shown to reduce burnout symptoms in special education teachers and physicians.

-

Design & Procedures

Describe the study, providing detail regarding the study intervention (drug, device, physical procedures, manipulation of the subject or the subject's environment, etc.). Discuss justifications for placebo control, discontinuation or delay of standard therapies, and washout periods if applicable. Identify procedures, tests and interventions performed exclusively for research purposes or more frequently than standard of care. Include alternative therapies, concurrent therapies discontinued per protocol, risk benefit ratio, and use of tissue/specimens. Discuss monitoring during washout periods if applicable. Include brief description of follow-up, if any.

This is a single-blind, randomized, controlled trial (RCT) of TM (N=64) vs. Control (CTL, N=64) in frontline HCPs. HCPs will be screened by a single-item stress scale and Columbia Suicide Severity Rating Scale (CSSRS). The Global Severity Index of the Brief Symptom Inventory (BSI)-18 Global Severity score will be used as the primary outcome for pre- and post-TM training (baseline, 1 vs. 3 months).

Study activities:

1. Introductory informational session at baseline visit (by remote) after informed consent has been obtained: Potential participants watch a video describing key points about TM, including opportunity for Q & A (by videoconference), so they will have a clear understanding of the TM technique and how it works. Anoptional recording of the information session will be available for those tha cannot meet the times of the live sessions.
2. Baseline testing including all psychological and physiological measures, as well as demographic and medical history forms:
    1. Brief Symptom Inventory (BSI)-18 Global Severity Index
    2. Maslach Burnout Scale (MBI)
    3. PHQ-9 (depression)
    4. GAD-7 (anxiety)
    5. Insomnia Severity Index (ISI)
    6. Connor Davidson Resilience Scale-25 (CD-RISC)
    7. Baseline HRV and GSR
    8. Subjective units of distress scale (SUDS)
    9. Baseline fMRI for those in the fMRI sub-groups
    10. Demographic (age, race, ethnic origin, marital status, education, living arrangements, number of children, religious affiliation, and employment status) and medical history (Previous medical history, ongoing medical issues, social history-alcohol/tobacco /recreational drug use, current medications use
3. Eligibility confirmation and randomization (eligibility checklist will be completed by the study coordinator (SC) and sent to statistician who will double-check eligibility and randomize subjects to treatment groups)
4. SC contacts participants to inform them of treatment assignment
5. Participants assigned to TM arm are contacted by TM instructor to set up times for the instruction sessions (described below).
6. Core TM instruction: There are 5 Core TM Instruction sessions (Sessions 1-5). Sessions 2-5 are on four consecutive days; each session is approximately 75 minutes:
    1. Core Session 1: Introductory/Preparatory session—Review of previous scientific research on the TM program, discussion of the mechanics and origin of the TM technique, personal Interview with a certified TM teacher
    2. Core Session 2: Personal instruction: individual one-on-one instruction in the TM technique
    3. Core Session 3: Day 1 of Verification of correct practice and further instruction (group session)
    4. Core Session 4: Day 2 of Verification of correct practice and review of the mechanics of the TM technique based on personal experiences (group session).
    5. Core Session 5: Day 3 of Verification of correct practice; discussion of the development of human potential and optimal wellness (group session).
7. TM arm Follow-up Sessions, to verify and support correct practice of the technique (group sessions, 45-minutes; can be done by remote):
    1. Follow-Up Session 1 ("10-Day Check"): occurs 10 days following the conclusion of the Core Instruction
    2. Follow-Up Session 2: occurs two weeks thereafter
    3. Follow-Up Sessions 3-4: occur at 3-week intervals following Follow-Up Session 2.

Participants randomized to the TM intervention will be asked to complete a short questionnaire to selfreport home TM completion for compliance and for comparison with the app collected data.
Duration of study period is 3 months. Last Follow-Up Session will be in the final week of Month 3. Wait-list controls (only 32) will be eligible to learn TM at the conclusion of the study period.

Physiological testing (baseline/3 months):

Heart Rate Variability (HRV) - Participants will be asked to wear an Apple Watch or Empatica device throughout trial participation to assess changes in resting HRV, during meditative practice, EKG and oxygen saturation will be measured on the apple watch or empatica device.

Galvanic Skin Response (GSR) - A portable GSR device ((“e-Sense Skin Response” device by Mindfield Biosystems) or Empatica devic will be used to evaluate GSR score at baseline, in response to a neutral script (generated by participant), then in response to the stressful script(generated by participant). Two electrodes, one each on index and middle fingers, will be attached to one hand. Once the data has been collected according to this protocol, the data will be uploaded from the subject’s smart phone via app and stored securely by Pattern health. It will be downloaded in REDCap database for statistical analysis.
Neuro-function (fMRI): Blood oxygenation level dependent (BOLD) fMRI is a robust and well tested method for tracking brain activity. BOLD fMRI tracks relative increases in blood oxygenation levels in the brain which can be used as secondary indicators of neural activity. Since local blood flow is a direct consequence of neurotransmitter action, BOLD fMRI can be used to track neural activity. A 3 T Siemens Trio Scanner will be used to acquire images. A five-minute magnetization prepared, rapid acquisition gradient echo image (MPRAGE) was used to do functional data and spatial normalization anatomic overlays (T1 MN1 Template). BOLD imaging used a 33-slice whole-brain, single-shot gradient echo (GE) echo-planar (EPI) sequence (TR/TE = 2000/25 ms, FOV = 240 mm, matrix = 64 × 64, lice thickness/gap = 4/0 mm). This sequence delivers a nominal voxel resolution of 3 × 3 × 3 mm.

fMRI Protocol: The first 32 eligible (exclusion criteria: pregnancy, claustrophobia, reluctance to participate) patients from each group will be randomly assigned to get fMRI at baseline and at the end of the study.

Subjects will meet for one hour with neuropsychiatrist or delegated trained staff who will evaluate the patient’s stressful event, obtain a full description of the event for presentation back to the patient as a script, and assess the patient’s reactivity to that event. Each participant will be interviewed to generate a written script of his or her distressing pandemic-related recollection and a separate script of a neutral recollection (i.e. getting up in the morning). It is a standard psychophysiological research method to use scripts as triggering cues for eliciting traumatic stress responses. Further, it is noted that in persons with a prolonged, intense memory of an event, it is unlikely that describing the event in the assessment session will have a significant impact on autonomic arousal or emotional reactivity to the event thereafter. Next, electrodes will be attached on one hand and bilaterally to the wrists to measure Galvianc Skin Response (GSR). The subject will be instructed to sit quietly with eyes closed without moving until stabilization of the heart rate occurs. Data recordings will begin with a one-minute period of visualizing a neutral event. After a 2-minute recovery period, subjects will be asked to recall and attempt to re-experience the feelings associated with their distressing illness-related image. Details previously provided by the subject will be used to prompt this condition. Subjects who demonstrate autonomic reactivity to the distressing image, as defined by an increase in HR of at least 5% (about 4 bpm) and/or at least 33% increase in skin conductance when the distressing event is compared to the neutral event, will qualify for the fMRI study. This also demonstrates the fidelity of the scripts in evoking the appropriate physiological response to the traumatic memory. These changes will also be recorded and used as a covariate during the MRI analysis.

MR data will be acquired using a GE Premiere UHP 3.0T MR scanner. The scanner has simultaneous multislice (multiband) capability which enables BOLD data acquisition to be obtained at high temporal resolution. The scanning methods and the protocol at each time point will be identical. Auditory stimuli will be presented to the subject during fMRI using a Resonance Technology Serene Sound system with MRcompatible headphones. The imaging protocol is designed to allow for acquisition of all scans in one session lasting less than 1 hour.
. A high-fidelity headphone will be placed over the subject’s ears for operator-subject communication, and for the subject to hear the audio file played during the neutral and trauma event BOLD scans. The imaging protocol will include the following scans: 1. 3 plane localizer (30 s) that provides anatomic localization of the head; 2. High resolution 3D T1-weighted anatomic scan (MP-RAGE, 6 minutes, TR/TE/ɑ = 1600ms/2. 46ms/9o, 0.9mm3 isotropic voxels, 176 contiguous sagittal slices of 1mm thickness, NEX=2); 3. Gradient Echo (GRE) field map (2.5 minutes,) for mapping of magnetic field inhomogeneities that can be used to unwarp the acquired functional volumes during data preprocessing; 4. Multi band Resting-state BOLD (10 minutes, TR/TE/ɑ = 1s/25ms/90o) during which the subject will be instructed to close eyes, relax, and to not think about anything in particular; 5. BOLD fMRI With Trauma Presentation will have subjects undergo two additional sessions with BOLD fMRI during a

neutral condition and while being exposed to an audio script of the stressful memory (each scan will be 5 minutes long). The subjects will first undergo the neutral scan during which they listen to an audio file of a neutral autobiographical account of their morning activities (i.e. getting up, eating breakfast). This neutral stimulus is designed to eliminate auditory stimulation that occurs during the traumatic memory task, and also provides a script with autobiographical content. Then subjects will undergo the task in which they listen to an audio file of the script of their most stressful memory related to taking care of patients during the COVID pandemic. The order of the presentation of these two scans will be randomly determined for each sub. The scripts are all read by the neuropsychiatrist who obtained the scripts from the patient. This standardizes the readings, voice, and tone of each script, both neutral and stressful, for every subject

Mobile app developed by Pattern Health Technologies, Inc. (PHT), an adherence and behavior change platform company that develops mobile technology and interfaces with wearable devices will administer and collect information from clinical questionnaires (BSI-18, MBI, PHQ-9, GAD-7, ISI, and CD-RISC) at prescribed intervals (baseline, 1 months, 3 months) and will provide daily push notifications of encouragement. The app will be accessed on Apple Watch or or Empatica device that will be given to the participants for the study. The Empatica is a medical-grade wearable devices that offers real-time physiological data acquisition.
Conduct a feasibility study of the proposed mobile app (Pattern Health Technologies, Inc) and Apple Watch or or Empatica device. Up to 10 healthy volunteers after consent, will utilize app and questionnaires as well as the apple watch or or Empatica device for heart rate variability. Formal written feedback will be obtained on user friendliness as well as gap in functionality. This feedback will be utilized for refining and optimization of app development.

Participants will be given a weekly resource and reminder guide to help them with adherence to study activities. This will be distributed via email.

Selection of Subjects

- List inclusion/exclusion criteria and how subjects will be identified.

Inclusion Criteria:

1. Full-time healthcare workers providing patient care currently during the COVID-19 pandemic (including physicians, trainee physicians, nurses, nurse practitioners, Physician Assistants, respiratory therapists, nurse assistants (NA), pharmacists, pharmacy technicians, occupational therapists, Radiology, neurology and cardiology clinical technicians/ technologist, Pulmonary Function technologists, Speech pathologists, Audiologists, Physical therapists, Exercise physiologists, Exercise specialist, Dietitian, Social worker, Patient health educator, Health Unit Coordinator, Nuclear med technician, Occupational Health technician, Certified medical assistant, Phlebotomy technician, Radiation therapist, Radio pharmacists, Vascular technician, Anesthesia technician, Behavior Health Technician, Cardiac sonographer, Podiatrist, Sonographer, Surgical technician, front desk staff, research/clinical healthcare workers without credentials, optometrists, emergency responders, EMS, dental assistants, orthodontists, endodontists, dentists, and dental hygienists.
2. A single-item stress scale will be used as a screen for eligibility; a minimum score of 6 on a 10point response scale will be needed to meet inclusion criteria.
3. Subjects who have at least a 5% increase from baseline in heart rate after exposure to a personalized stressful script OR at least a 33% increase in skin conductance after exposure to the script.
4. Willingness to address burnout symptoms by non-pharmacological means.
5. All subjects must provide Informed Consent prior to enrollment in the study.
6. Willingness to wear the provided Apple Watch or or Empatica device for the data collection process.

Exclusion Criteria:

1. Anti-psychotic medications or beta-blockers
2. Current suicidal or homicidal ideation (suicidal ideation as screened by C-SSRI survey preenrollment). If positive, subjects will be sent to PCP or health care provider.
3. Previous instruction in TM

Subject Recruitment and Compensation

Describe recruitment procedures, including who will introduce the study to potential subjects. Describe how you will ensure that subject selection is equitable and all relevant demographic groups have access to study participation (per 45 CFR 46.111(a) (3)). Include information about approximately how many DUHS subjects will be recruited. If subjects are to be compensated, provide specific prorated amounts to be provided for expenses such as travel and/or lost wages, and/or for inducement to participate.

The target population is Health care Providers (HCPs). For this study purpose, we define HCPs as Physicians, Physician Trainees, Physician Assistants, Nurse Practitioners, Nurses, Respiratory Therapists, nurse assistants (NA), pharmacists, pharmacy technicians, occupational therapists, Radiology, neurology and cardiology clinical technicians/ technologist, Pulmonary Function technologists, Speech pathologists, Audiologists, Physical therapists, Exercise physiologists, Exercise specialist, Dietitian, Social worker,
Patient health educator, Health Unit Coordinator, Nuclear med technician, Occupational Health technician, Certified medical assistant, Phlebotomy technician, Radiation therapist, Radio pharmacists, Vascular technician, Anesthesia technician, Behavior Health Technician, Cardiac sonographer, Podiatrist,
Sonographer, Surgical technician, front desk staff, research/clinical healthcare workers without credentials involved in patient care during COVID-19, optometrists, emergency responders, EMS, dental assistants, orthodontists, endodontists, dentists, and dental hygienists. We aim to recruit from the Duke University Health System that has more than 1000 Physician trainees, approximately 2000 physicians and a proportional number of other HCPs. Durham Veterans Affairs Health center HCPs will be eligible to participate at the Duke Site as well. This is a diverse study population that will truly represent HCPs in the VA, military and civilian healthcare workforce. The recruitment will be by: Flyers, word of mouth and informing via email to stakeholders such as medical staff offices, respiratory care services, newsletters.

We aim to recruit up to 128 volunteer HCPs after screening with inclusion and exclusion criteria. The study will be open to both male and female HCPs. Recruitment will be via Duke lRB-approved local advertising (flyers), Facebook social media, blast email and by colleague referrals from Duke HCPs. We plan to recruit from Duke, Durham VA, UNC, Rex, and Wake Med, local private practice including HCP's in primary care, internal medicine, pediatrics, and specialty care.

Once potential participants complete the pre-screen survey and express interest, we will use a Google messaging account and / or RedCap Twillio SMS messaging in order to reach out to these potential participants if needed. By using text messaging, the pre-screen to screening period will be completed in a more timely manner to begin enrollment.

Up to 10 healthy volunteers for feasibility study of the proposed mobile app (Pattern Health Technologies, Inc) and Apple Watch, will be recruited from Pro00102890.

There is no compensation for participation, but those that complete the 3 month study participation may keep the apple watch used in the study.

Up to 10 participants will be enrolled at the Emory site, piloting the study at a second location, 5 Tm and 5 TAU.

Consent Process

- Complete the consent section in the iRIS Submission Form.

Subject's Capacity to Give Legally Effective Consent

- If subjects who do not have the capacity to give legally effective consent are included, describe how diminished capacity will be assessed. Will a periodic reassessment occur? If so, when? Will the subject be consented if the decisional capacity improves?

All subjects must be able to give legal effective consent. The ability to consent is one of the inclusion / exclusion criteria.

Study Interventions

If not already presented in #4 above, describe study-related treatment or use of an investigational drug or biologic (with dosages), or device, or use of another form of intervention (i.e., either physical procedures or manipulation of the subject or the subject's environment) for research purposes.

The Emory site is closed to accrual but has active participants in follow up expected to finish this month. The Emory IRB deemed that no continuing review was required so did not provide an expiration date for the study at that site. See the comments below from the Emory approval document:

"On 3/15/2021 the Emory IRB reviewed the above-referenced study by expedited process. This research is eligible for expedited review under 45 CFR.46.110 and/or 21 CFR 56.110 because it poses minimal risk and fits expedited review category F7 as set forth in the Federal Register. No annual IRB review is required, as permitted under the 2018 Common Rule."

Risk/Benefit Assessment

- Include a thorough description of how risks and discomforts will be minimized (per 45 CFR 46.111(a) (1 and 2)). Consider physical, psychological, legal, economic and social risks as applicable. If vulnerable populations are to be included (such as children, pregnant women, prisoners or cognitively impaired adults), what special precautions will be used to minimize risks to these subjects? Also identify what available alternatives the person has if he/she chooses not to participate in the study. Describe the possible benefits to the subject. What is the importance of the knowledge expected to result from the research?

Risk: There is no more than minimal risk for this study

Benefit: There is a marked paucity of data for interventions and especially mediation interventions as potentially efficacious therapeutics in HCP burnout despite the urgent and acute clinical need due to the COVID-19 crisis. The results of this proposed trial could thus provide the scientific foundation for the potential efficacy of Transcendental Meditation.

Protection against Risk
a) *Physical Risk and Participant Safety*: As mentioned above other than skin irritation by Apple watch or Empatica device, we do not anticipate any physical risk.

b) *Emotional Distress and Participant Safety*: The intervention is not known to cause emotional distress but to be proactive all participants will be carefully assessed before the study and will be made aware of psychiatric emergency services. During the informed consent process, participants will be advised that the study procedures could potentially lead to distress and that they may withdraw from the study at any time. If a participant withdraws from the study, Dr. Lee and Dr. Joshi will be immediately available by cell phone at all times for any concerns regarding potential worsening of medical symptoms or the emergence of suicidal or homicidal ideation. Should any emergent issues arise that require immediate medical and/or psychiatric attention, the Duke Medical center has fully staffed Emergency Department and Psychiatric Emergency Care (PEC) services which provide acute medical and psychiatric care 24 hours per day 7 days per week. In terms of risks to confidentiality, pertinent information regarding potential harm, including suicidal and homicidal intent will be shared as necessary and required by law with clinicians and/or the appropriate authorities. In such circumstances, records may be made available to authorities, even without the participant's consent.

c) *Unauthorized Access to Data:* There is a possibility of an unauthorized party gaining access to secure PHI contained in the database. This is also a low risk, and several precautions have been implemented to minimize unauthorized access. First, reports from participants' clinical records concerning research observations will not be made available to outside medical facilities without the written consent of the participant. All clinical and biometric data obtained from research interviews and the laboratory will be coded. The data will be kept in locked file cabinets and accessible only to authorized research personnel. Only study numbers will appear on specimens,

data and documents used for evaluation or statistical analysis. In addition, any publications resulting from this research will not identify individual participants. Subjects who utilize the e-consent process are sent an e-mail copy of the consent form. Email is not a secure method of communication so a risk of loss of privacy and confidentiality. d) *Risks of fMRI*
Magnetic resonance imaging (fMRI) uses a magnet and radio waves to make diagnostic medical images of the body. There have been no ill effects reported from exposure to the magnetism or radio waves used in this test. However, it is possible that harmful effects could be recognized in the future. A known risk is that the magnet could attract certain kinds of metal. Therefore, we will carefully ask participants about metal within their body, including medical implants, devices such as pacemakers and internal defibrillators, or certain dyes found in tattoos. We will also keep the examining room locked so that no one carrying metal objects can enter while participants are in the scanner.

If there is any question about potentially hazardous metal within your body, you will not undergo the MRI.

e) *Risks for Study Personnel:* There is minimal risk involved to study personnel

Potential Benefits of Research:
Benefits for Participants: Participants may experience an acute or perhaps short-term reduction of symptoms of Burnout. Alternatively, study participants may not receive benefits from the proposed research other than one Apple watch or or Empatica device; their participation may lead to a better understanding of HCP Burnout symptomatology and interventions. In terms of benefit to others, knowledge gained from the study may inform further management of HCP burnout and in turn lead to improved quality of care for patients.

Costs to the Subject

- Describe and justify any costs that the subject will incur as a result of participation; ordinarily, subjects should not be expected to pay for research without receiving direct benefit.

There are no additional costs to the subjects.

Data Analysis & Statistical Considerations

- Describe endpoints and power calculations. Provide a detailed description of how study data will be analyzed, including statistical methods used, and how ineligible subjects will be handled and which subjects will be included for analysis. Include planned sample size justification. Provide estimated time to target accrual and accrual rate. Describe interim analysis including plans to stop accrual during monitoring. Phase I studies, include dose escalation schema and criteria for dose escalation with definition of MTD and DLT.

For all primary and secondary outcomes, data will be analyzed using the method of analysis of covariance (ANCOVA) with repeated measures at each battery of measurements for psychological distress, burnout, anxiety, depression, insomnia, resilience and blood rate variability-related heart diseases. Dependent variables will be changed from baseline at three-month and at six-month post-test with treatment group as the independent variable and baseline characteristics as covariates. Effect sizes will be calculated using Cohen's d: mean differences divided by the pooled standard deviation. In addition, the responder's analysis will be performed. A participant is defined as a responder if the participant meets a pre-specified criteria (50% improvement or greater) of improvement post-treatment at 1 month or at 3 months. Exploratory analyses such as subgroup analyses may be performed as deemed appropriate by the principal investigators or biostatisticians. For the primary outcome, change in total burnout, the significance level will be set at $p<0.05$, two-tailed.

Data & Safety Monitoring

- Summarize safety concerns, and describe the methods to monitor research subjects and their data to ensure their safety, including who will monitor the data, and the frequency of such monitoring. If a data monitoring committee will be used, describe its operation, including stopping rules and frequency of review, and if it is independent of the sponsor (per 45 CFR 46.111(a) (6)).

In accordance with federal regulations the PI will monitor for, review, and promptly report to the IRB, appropriate institutional officials, sponsor, coordinating center and the appropriate regulatory agency head all unanticipated problems involving risks to subjects or others that occur in the course of a subject's participation in a research, all AE reports will be reported per the DUHS IRB policies.

Privacy, Data Storage & Confidentiality

Complete the Privacy and Confidentiality section of the iRIS submission form.

Describe Role of External Personnel:

The Neuropsychological staff, or trained study staff, will facilitate the administration of some of the study questionnaires as delegated by their role. Dr. Sandeep Vaishnavi, (external KP) will administer some of the questionnaires and train other study staff in the administration the questionnaires. Dr. Vaishnavi will not have access to PHI during his participation as external KP on the study until a fully executed agreement is in place. His agreement is now fully executed so Dr. Vaishnavi will interact with participants to administer SUDS, CSSR, and complete stressful and neutral script generation.

Data will be acquired by the Emory site through the pattern Health app then analyzed by the study statistician at Emory. Duke will purchase the Apple Watches used for the study, and send 10 to the Emory site, so Duke staff will have a record of the serial numbers of the watches given to the Emory participants, but will not know which participant received specific watches.

Study Scope

Does this study have a cancer focus? Cancer focus includes studies that enroll >50% oncology or malignant hematology patients; or, preventing, detecting, and diagnosing cancer or understanding the impact of cancer on patients and their caretakers.

○ Yes ◉ No

Are you using a drug, biologic, food, or dietary supplement in this study?

○ Yes ◉ No

Are you using a medical device, an algorithm (whether computer based or not), an in vitro diagnostic test, or using samples to look for biomarkers in this study?

○ Yes ◉ No

Does this study employ magnetic resonance, including imaging (MRI), spectroscopy (MRS), angiography (MRA) or elastography (MRE) beyond the standard of care?

◉ Yes ○ No

Does this study specify or require the performance of diagnostic procedures using ionizing radiation (x-rays, DEXA, CT scans, nuclear medicine scans, etc.) that are beyond the standard of care?

○ Yes ◉ No

Does this study specify or require the performance of therapeutic procedures using ionizing radiation (accelerator, brachytherapy or systemic radionuclide therapy) that are beyond the standard of care?

○ Yes ◉ No

Will the participant be subjected to increased or decreased ambient pressure?

○ Yes ◉ No

Do you plan to recruit subjects from Duke Regional Hospital (DRH)?

○ Yes ◉ No

Do you plan to recruit subjects from Duke Raleigh Hospital (DRAH)?

○ Yes ◉ No

Does this study utilize the Duke Early Phase Clinical Research Unit (DEPCRU)?

( ) Yes (•) No

Are you using the Duke logo in any advertisements?

( ) Yes (•) No

Is this study retrospective, prospective, or both?

"Retrospective" means that data or samples already in existence (collected prior to the study submission) will be used.
"Prospective" means there will be data or samples collected in this study for research purposes.

( ) Retrospective
(•) Prospective
( ) Retrospective and Prospective

If the study is both retrospective and prospective: Is this a review soley of information collected for non-research purposes (i.e. a review of medical records)?

( ) Yes ( ) No

Does this protocol include any research using botulinum toxin, including the FDA-approved clinical product (Botox)?

( ) Yes (•) No

Does this protocol involve the administration of any of the following materials to humans?

- Any viral vector or plasmid
- Any cells that have been modified by a viral vector
- Any other genetically-modified cells
- Any genetically-modified virus, bacterium, or other agent
- Any other recombinant or synthetic nucleic acid

( ) Yes (•) No

Subject Population Groups and Enrollment

Population Groups (Select targeted population groups only):

Note:

- Students and Employees over whom Key Personnel have a supervisory role may not be enrolled in this study.

[x] Adults
[ ] Minors who are Wards of State
[ ] Minors
[ ] Duke Patients
[x] Pregnant Women
[ ] Fetuses
[ ] Prisoners
[ ] Adults incapable of giving consent
[ ] Adults with diminished capacity
[ ] Handicapped subjects

☑ Employees
☑ Healthy Controls
☐ Deceased subjects
☐ Blanket Protocol

Students and Employees over whom Key Personnel have a supervisory role may not be enrolled in this study.

Please select any population groups excluded from participation in this study:

☐ Pregnant women

Maximum number of subjects to be consented at Duke:

Enter a single number. If you anticipate consenting a range of subjects, enter the **upper** limit of the range. The number should represent the maximum number of subjects for the life of the study.

138

Maximum number of subjects to be consented at all sites:

Enter a single number. If you anticipate consenting a range of subjects, enter the **upper** limit of the range. The number should represent the maximum number of subjects for the life of the study.

138

Subject Procedures and Costs

Biobank - Does this study involve the collection, use, tracking, banking (storage) or distribution of human biological specimens?

Human biological specimens include blood or its components, healthy or diseased tissue, bodily fluids, DNA /RNA or human stem cells.

○ Yes ◉ No

Procedures

Check all the apply:

☐ Genetic Testing
☐ Gene Transfer
☐ DNA Banking
☐ Testing for Reportable Infectious Diseases
☐ Human Cell Banking
☐ *Use of Human Embryonic Stem Cells
☐ *Use of Human-induced Pluripotent Stem Cells
☐ *Use of Other Cells Derived from Human Embryos
☐ *Use of Human/Animal Chimeric Cells
☐ *Specialized Cell Populations for Cell Therapy
☐ Use of Human Tissue
☐ Use of Bodily Fluids
☐ Use of Blood (or its components)
☐ [illegible]

Will blood be drawn in this study for research purposes?

○ Yes ◉ No

Will the Operating Room be used in this study?

Include only research time, not clinical care time.

○ Yes ◉ No

Will there be extra costs to subjects or insurance as a result of the research (e.g. tests, hospitalization)?

○ Yes ◉ No

Will there be Subject Compensation?

◉ Yes ○ No

Compensation for Travel / Lost Income (in USD):

0

Other Subject Compensation:

Participants will be able to keep the wearable device (apple watch)used in the study collection process, after completion of the study.
Participants that are non-Duke staff that have a fMRI scheduled as part of study participation will be provided a parking pass for each MRI encounter.

Subject Recruitment Materials

For each document to be reviewed, use the table below to provide the following information:

Attach a copy of each advertisement that you will be using with this study in the Initial Submission Packet. If any Ad will have multiple wording variations, attach a copy of each version of the Ad.
All materials that will be used to advertise the study in order to recruit subjects must be approved by the IRB.

Types of subject recruitment materials include, but are not limited to, the following:

Direct Advertising
Posters
Billboards
Flyers
Brochures

Media Advertising
Newspaper Ads
Magazine Ads
Radio Ads
TV commericals / Video
Internet website
Social Media

Newsletter
Email
Postcards / Letters

(Note: Doctor-to-Doctor letters do not require IRB approval)

Document name	Material category	Location material displayed	Has this material previously been approved by the IRB?
HCP Flyer	(•) Billboard / Flyer / Poster<br>( ) Brochure<br>( ) Internet website / Email<br>( ) Letter / Postcard<br>( ) Phonescript<br>( ) Radio<br>( ) Television / Video<br>( ) Newsletter / Newspaper / Magazine<br>( ) Other	Please be specific. For example, "Duke" would not be an appropriate location. "Duke Hospital Television" would be an appropriate response.<br>Cork board, Faculty lounge, GME office, DAAAC, health care provider gathering areas, Hanes House	( ) Yes (•) No
Blast email to HCP	( ) Billboard / Flyer / Poster<br>( ) Brochure<br>(•) Internet website / Email<br>( ) Letter / Postcard<br>( ) Phonescript<br>( ) Radio<br>( ) Television / Video<br>( ) Newsletter / Newspaper / Magazine<br>( ) Other	Please be specific. For example, "Duke" would not be an appropriate location. "Duke Hospital Television" would be an appropriate response.<br>email to HCPs	( ) Yes (•) No
Email Recruitment invite for TM Intro Talk	( ) Billboard / Flyer / Poster<br>( ) Brochure<br>(•) Internet website / Email<br>( ) Letter / Postcard<br>( ) Phonescript<br>( ) Radio<br>( ) Television / Video<br>( ) Newsletter / Newspaper / Magazine<br>( ) Other	Please be specific. For example, "Duke" would not be an appropriate location. "Duke Hospital Television" would be an appropriate response.<br>Blast email	( ) Yes (•) No
	( ) Billboard / Flyer / Poster<br>( ) Brochure<br>(•) Internet website / Email<br>( ) Letter / Postcard	Please be specific. For example, "Duke" would not be an appropriate location. "Duke Hospital Television" would be	

Email to Div Chiefs	Phonescript<br>○ Radio<br>○ Television / Video<br>○ Newsletter / Newspaper / Magazine<br>○ Other	an appropriate response.<br><br>Blast email to Div. Chiefs	○ Yes ◉ No
VA Flyer	◉ Billboard / Flyer / Poster<br>○ Brochure<br>○ Internet website / Email<br>○ Letter / Postcard<br>○ Phonescript<br>○ Radio<br>○ Television / Video<br>○ Newsletter / Newspaper / Magazine<br>○ Other	Please be specific. For example, "Duke" would not be an appropriate location. "Duke Hospital Television" would be an appropriate response.<br><br>Cork board, Faculty lounge, bulletin boards in the Durham VA facility	○ Yes ◉ No
phone script for pre-screening	○ Billboard / Flyer / Poster<br>○ Brochure<br>○ Internet website / Email<br>○ Letter / Postcard<br>◉ Phonescript<br>○ Radio<br>○ Television / Video<br>○ Newsletter / Newspaper / Magazine<br>○ Other	Please be specific. For example, "Duke" would not be an appropriate location. "Duke Hospital Television" would be an appropriate response.<br><br>Phone script for study staff to prescreen potential participants (the second half of the redcap prescreen form)	○ Yes ◉ No
recruitment in newsletter	○ Billboard / Flyer / Poster<br>○ Brochure<br>○ Internet website / Email<br>○ Letter / Postcard<br>○ Phonescript<br>○ Radio<br>○ Television / Video<br>◉ Newsletter / Newspaper / Magazine<br>○ Other	Please be specific. For example, "Duke" would not be an appropriate location. "Duke Hospital Television" would be an appropriate response.<br><br>newsletter to Duke employees	○ Yes ◉ No
	○ Billboard / Flyer / Poster<br>○ Brochure<br>◉ Internet website / Email	Please be specific. For example, "Duke" would not be an	

ZOOM invite e-mail	○ Letter / Postcard<br>○ Phonescript<br>○ Radio<br>○ Television / Video<br>○ Newsletter / Newspaper / Magazine<br>○ Other	"Duke Hospital Television" would be an appropriate response.<br>Email to potential participants	○ Yes ◉ No
Webinar registration	○ Billboard / Flyer / Poster<br>○ Brochure<br>○ Internet website / Email<br>○ Letter / Postcard<br>○ Phonescript<br>○ Radio<br>○ Television / Video<br>○ Newsletter / Newspaper / Magazine<br>○ Other	Please be specific. For example, "Duke" would not be an appropriate location. "Duke Hospital Television" would be an appropriate response.<br>registration for webinar study information session	○ Yes ◉ No
Informational webinar	○ Billboard / Flyer / Poster<br>○ Brochure<br>○ Internet website / Email<br>○ Letter / Postcard<br>○ Phonescript<br>○ Radio<br>○ Television / Video<br>○ Newsletter / Newspaper / Magazine<br>◉ Other	Please be specific. For example, "Duke" would not be an appropriate location. "Duke Hospital Television" would be an appropriate response.<br>Webinar for information about TM to potential participants	○ Yes ◉ No
Recoding of webinar	○ Billboard / Flyer / Poster<br>○ Brochure<br>○ Internet website / Email<br>○ Letter / Postcard<br>○ Phonescript<br>○ Radio<br>◉ Television / Video<br>○ Newsletter / Newspaper / Magazine<br>○ Other	Please be specific. For example, "Duke" would not be an appropriate location. "Duke Hospital Television" would be an appropriate response.<br>Recording of Webinar for information about TM to potential participants	○ Yes ◉ No
	○ Billboard / Flyer / Poster<br>○ Brochure<br>◉ Internet website / Email	Please be specific. For example, "Duke" would not be an	

Invite for recording of Webinar	◯ Phonescript<br>◯ Radio<br>◯ Television / Video<br>◯ Newsletter / Newspaper / Magazine<br>◯ Other	"Duke Hospital Television" would be an appropriate response.	◯ Yes ◉ No

Consent Process

Attach draft consent forms in the Initial Review Submission Packet.

Consent forms must be MS Word documents and follow the specific format outlined by the IRB. **Click here** to download a copy of the consent form template.

Note: Please do not edit the section of the footer that contains the Protocol ID, Continuing Review and Reference Date fields. Those fields will be used to stamp the final consent form when it is approved by the IRB. If you want to add an internal version date, please put it in the header.

Who will conduct the consent process with prospective participants?

Give the person's role in this study (PI, Study Coordinator, etc.):

Clinical Research Coordinator

Who will provide consent or permission?

(Select all that apply):

- [x] Participant
- [ ] Parent(s) or Legal Guardian(s)
- [ ] Legally Authorized Representative (LAR)

How much time will the prospective participant (or legally authorized representative) have between being approached about participating in the study and needing to decide whether or not to participate?

If you are not giving the person overnight to consider whether or not to participate, please justify.

As much time as needed

Where will the consent process occur?

The consent process will occur in a private clinic room at the Duke Asthma, Allergy and Airway Center or via eConesnt.

What steps will be taken in that location to protect the privacy of the prospective participant?

N/A - protections are already in place in clinic rooms. IF performing via e-consent the participant will be reminded to complete in a private location.

How much time will be allocated for conducting the initial consent discussion, including presenting the information in the consent document and answering questions, with each prospective participant?

As much time as needed

What arrangements will be in place for answering participant questions before and after the consent is signed?

The subject will be given the study coordinator's and PI's contact information for any additional questions.

Describe the steps taken to minimize the possibility of coercion or undue influence.

Subjects are told participation is voluntary and there decision to participate will not involve any penalty or loss of benefits

What provisions will be in place to obtain consent from participants who do not read, are blind or who do not read/understand English?

We will only consent subjects who are able to read consents in English on their own.

Do you plan to obtain written consent for the conduct of research?

(•) Yes ( ) No

Protected Health Information (PHI)

Indicate how you intend to use potential subjects' Protected Health Information (PHI):

( ) I will review, but not record, PHI prior to consent.
(•) I will record PHI prior to consent.
( ) I do not intend to use PHI prior to consent.
( ) I will record PHI without consent. (decedent research, database repository, chart review)

Request for Waiver or Alteration of Consent and/or HIPAA Authorization

Will the population include deceased individuals?

( ) Yes (•) No

This waiver request applies to the following research activity or activities:

- [ ] Scheduling of research activities in MaestroCare and/or the recording of PHI via telephone for screening purposes prior to obtaining written consent for the research. Scheduling of research activities in MaestroCare and/or the recording of PHI via telephone for screening purposes prior to obtaining written consent for the research. (If you check this box, please complete all sections below.)
- [x] Ascertainment (identification, selection) and/or recruitment of potential subjects while recording identifiable private information, such as protected health information (PHI), prior to obtaining the subject’s consent. (If you check this box, please complete sections B and C below.)
- [ ] Conduct of the research project without obtaining verbal or written consent and authorization. (If you check this box, please complete sections B and C below.)

Note: Answer the questions below as they pertain solely to PHI collected prior to consent.

Provide the following information:

List the elements of informed consent and/or HIPAA authorization for which waiver or alteration is requested:

- Provide the rationale for each.

Request waiver of the core elements of consent and HIPAA authorization in order to record protected health information (PHI) prior to obtaining the subject's consent for the purpose of ascertaining appropriateness of potential subjects.

List the specific protected health information (PHI) to be collected and its source(s):

- (Note: PHI = health information + identifiers)

Name, date of birth, contact information. The source of this PHI may be directly from the subject. The PHI collected prior to consent will be used to recruit and screen only. Only the minimum amount of information will be recorded. This information will not leave the covered entity.

Criteria for Waiver: The DUHS IRB may waive the requirement for informed consent and authorization if all of the following criteria are met:

- Please respond to each item in the space below using protocol-specific language to provide justification:

a) The research or clinical investigation involves no more than minimal risk to subjects:

Collection of PHI by study staff involves no more than minimal risk to potential subjects.

b) The waiver or alteration will not adversely affect the rights and welfare of the subjects. Include a description of any measures to be taken to ensure that the rights and welfare of subjects will be protected:

This waiver does not adversely affect the rights and welfare of subjects. In the event that the potential participant does not consent their information will be kept in a screening log only. After obtaining informed consent, the information will be protected according to usual standards of confidentiality applicable to research studies.

c) Whenever appropriate, the subjects will be provided with additional pertinent information after participation:

n/a

d) If this research activity relates to research involving deception, explain how subjects will be provided with additional pertinent information after study participation and what information will be provided. Otherwise indicate "not applicable":

n/a

e) The use or disclosure of protected health information involves no more than minimal risk to the privacy of individuals, based on, at least, the presence of the following elements (e1. and e2.)

Demonstrate that the use or disclosure of PHI involves no more than minimal risk to the privacy of subjects by describing the plans requested below:

e1) An adequate plan to protect the identifiers from improper use and disclosure.
Describe the plan (how protection will be accomplished) and indicate where the PHI will be stored and who will have access:

The PHI will be stored in the locked research office and in a secured password-protected computer system maintained at DUHS with limited access (key personnel only).

e2) An adequate plan to destroy the identifiers at the earliest opportunity consistent with conduct of the research, unless there is a health or research justification for retaining the identifiers or such retention is otherwise required by law.

Describe the plan (how and when identifiers will be destroyed and by whom). If there is a health or research justification for retaining the identifiers or such retention is otherwise required by law, provide the reason to retain identifiers:

If the potential subject enters the study, the PHI collected will become part of the subject's source documents. Potential subjects who do not sign the consent will have their information retained on a screening log.

e3) Adequate written assurances that the protected health information will not be reused or disclosed to any other person or entity except (i) as required by law, (ii) for authorized oversight of the research study, or (iii) for other research for which the use or disclosure of PHI would be permitted by the HIPAA Privacy Rule. By electronically signing this submission, the PI provides this written assurance:

ok

f) The research could not practicably be conducted or carried out without the waiver or alteration:

- Explain why informed consent/authorization can not be obtained from subjects.

It is impractical to obtain consent from potential subjects before ascertaining interest in participating.

g)The research could not practicably be conducted or carried out without access to and use of the protected health information:

Potential subjects could not be located, contacted and/or recruited without access to PHI.

h)For research using biospecimens or identifiable information, the research could not practicably be carried out without access to and use of the protected health information:

Potential subjects could not be located, contacted and/or recruited without access to PHI.

Privacy and Confidentiality

Explain how you will ensure that the subject's privacy will be protected:

Consider privacy interests regarding time and place where subjects provide information, the nature of the information they provide, and the type of experience they will be asked to participate in during the research.

The potential participants will either select a private location to perform e-consent or can have an in person consent at the Duke Asthma Allergy Airway Center in a private room.

Describe how research data will be stored and secured to ensure confidentiality:

How will the research records and data be protected against inappropriate use or disclosure, or malicious or accidental loss or destruction? Records and data include, for example, informed consent documents, case report forms or study flow sheets, survey instruments, database or spreadsheets, screening logs or telephone eligibility sheets, web based information gathering tools, audio/video/photo recordings of subjects, labeled specimens, data about subjects, and subject identifiers such as social security number.

Research records will be stored in REDCap and paper consent documents will be locked in a cabinet at the Duke Asthma Allergy Airway Center research office in a badge access secure location.

Please click Save & Continue to proceed to the Initial Submission Packet.

The Initial Submission Packet is a short form filled out after the protocol application has been completed. This is an area to attach protocol-related documents, consent forms, and review the application.

Intervention Protocol

Targeting the Health Care Worker (HCW) Burnout Crisis During the COVID-19 Pandemic with Meditation: A Randomized Clinical Trial

Transcendental Meditation Protocol

Core Session 1 included an overview of previous scientific research on the TM program, discussion of the mechanics and origin of the TM technique, and a personal interview with a certified TM teacher. Core Session 2 included personal instruction, with individual one-on-one instruction in the TM technique. Core Session 3 included verification of correct practice and further instruction, in a group session. Core Session 4 included verification of correct practice again and review of the mechanics of the TM technique based on personal experiences, in a group session. Core Session 5 included verification of correct practice and discussion of the development of human potential and optimal wellness, in a group session. Participants were instructed to practice TM twice daily during the 3 months study period. During this time, 4 additional TM follow-up sessions were scheduled to verify and support the correct practice of the technique (group sessions, 45-minutes; in-person or remote).

Follow-Up Session 1 (“10-Day Check”) occurred 10 days following the conclusion of the core Instruction. Follow-Up Session 2 occurred two weeks thereafter. Follow-Up Sessions 3 and4 occurred at 3-week intervals following Follow-Up Session 2.

SAP: Marcus Study

Created: 6/14/21
Last Updated: 3/21/22

Project: Targeting the Health Care Provider (HCP) Burnout Crisis during the COVID Pandemic

Investigators: Patty Lee (PI), Sangeeta Joshi, Sandeep Vaishnavi

Primary Statistician(s): Amanda Brucker
Faculty Statistician: Shein-Chung Chow

---

1. Background

This is a single blind pilot Randomized Clinical Trial (RCT) of TM (Transcendental Meditation) vs an active control of Treatment as Usual (TAU) in health care workers managing COVID-19 patients. A total of 80 subjects will be enrolled in this single-blind, randomized, controlled trial comparing TM (N=40) vs. TAU (N=40) in frontline healthcare providers (HCPs). This study will collect primary and secondary outcomes at baseline, 1 month, and 3 months, and additional measures continuously throughout the study (HRV, compliance).

2. Study Objectives

2.1 Specific Aims

1. The primary goal of this study is to evaluate the effectiveness of TM vs TAU to improve burnout symptoms in providers.
   - This will be assessed using the primary outcome of the BSI-18 questionnaire score which measures psychological distress. We will assess changes in the BSI-18 from baseline compared to 3 months.
2. Secondary goals are to compare changes in measures of burnout, insomnia, resilience, depression, and anxiety between the TM and TAU participants as measured in psychometric surveys. In addition, we will examine changes in HRV and neuro-functional features from baseline to 3 months.

2.2 Hypotheses

We hypothesize that

- Participants receiving the TM treatment will show a greater reduction in distress than those in the TAU group

3. Study Population

This study enrolled HCPs in the Durham, NC area currently providing patient care.

3.1 Additional Inclusion/Exclusion to apply to cohort

- Inclusions:
  - Willing to address burnout symptoms by non-pharmacological means
  - Willing to use a wearable device during the study
  - >=5% increase in HR OR >=33% increase in GSR between neutral and stressful script exercises at baseline visit
- Exclusions:

- Taking any anti-psychotic medications or beta-blockers at baseline
- Received previous instruction in TM
- <= 18 years at time of baseline visit
- Scores < 6 on SUDS at baseline visit
- Suicidal or homicidal ideation at baseline as assessed by C-SSRS

4. Study Endpoints

All endpoints will be compared between baseline and 3 months.

Outcomes:

- Primary
    - BSI-18 total score
- Secondary
    - BSI-18 subscores
    - MBI (burnout) and subscores
    - ISI (insomnia)
    - CD-RISC (resilience)
    - PHQ-9 (depression)
    - GAD-7 (anxiety)

5. Data

5.1 Data Storage

- Questionnaire data is stored in combination of Redcap and PatternHealth application database

5.2 Data Provenance

- Data were pulled from REDCap on 8/2/21 by Amanda Brucker
- PatternHealth application data was transferred through Box by Stephanie Gaillard & Jennifer McLaughlin and downloaded by Amanda Brucker on 6/22/21 and 8/2/21.

6. Statistical Analysis

For relevant analyses, significance will be assessed at level $\alpha=0.05$. All analyses will be performed using SAS 9.4. No missing data imputation will be applied and complete case analysis will be used. All summaries, comparisons, and analyses of the two treatment groups will follow the intent to treat (ITT) principle.

1. The flow of participants through the study will be represented in a CONSORT diagram (**Figure 1**).
2. Baseline demographics, medical history, and other characteristics will be summarized in the two treatment groups (**Table 1**). Continuous variables will be summarized with mean/standard deviation/median/Q1-Q3/min/max and categorical variables with frequency counts and percentages.
3. We will report the mean/standard deviation/median/Q1-Q3/min/max values of the psychometric endpoints at baseline and at 3m by treatment (**Table 2**).
    - In this analysis, we will also assess whether endpoints are normally distributed and whether there are any outliers. Any significant outliers will be discussed with the group.
4. We will also effect sizes for all psychometric endpoints using the Carlson-Schmidt method (**Table 3**).
5. We will employ general linear models to test for differences in changes in the psychometric endpoints between baseline and 3m by treatment group.

- Model fit estimates and 95% confidence intervals for the 3m-vs-baseline differences in the endpoints will be reported (**Table 4**). We will test for any difference in 3m-vs-baseline changes between the two treatment groups and report p-values.

6. Additional sensitivity, subgroup, and exploratory analyses will be performed following the completion of the main analysis.

7. List of Tables and Figures

Figure 1: CONSORT diagram

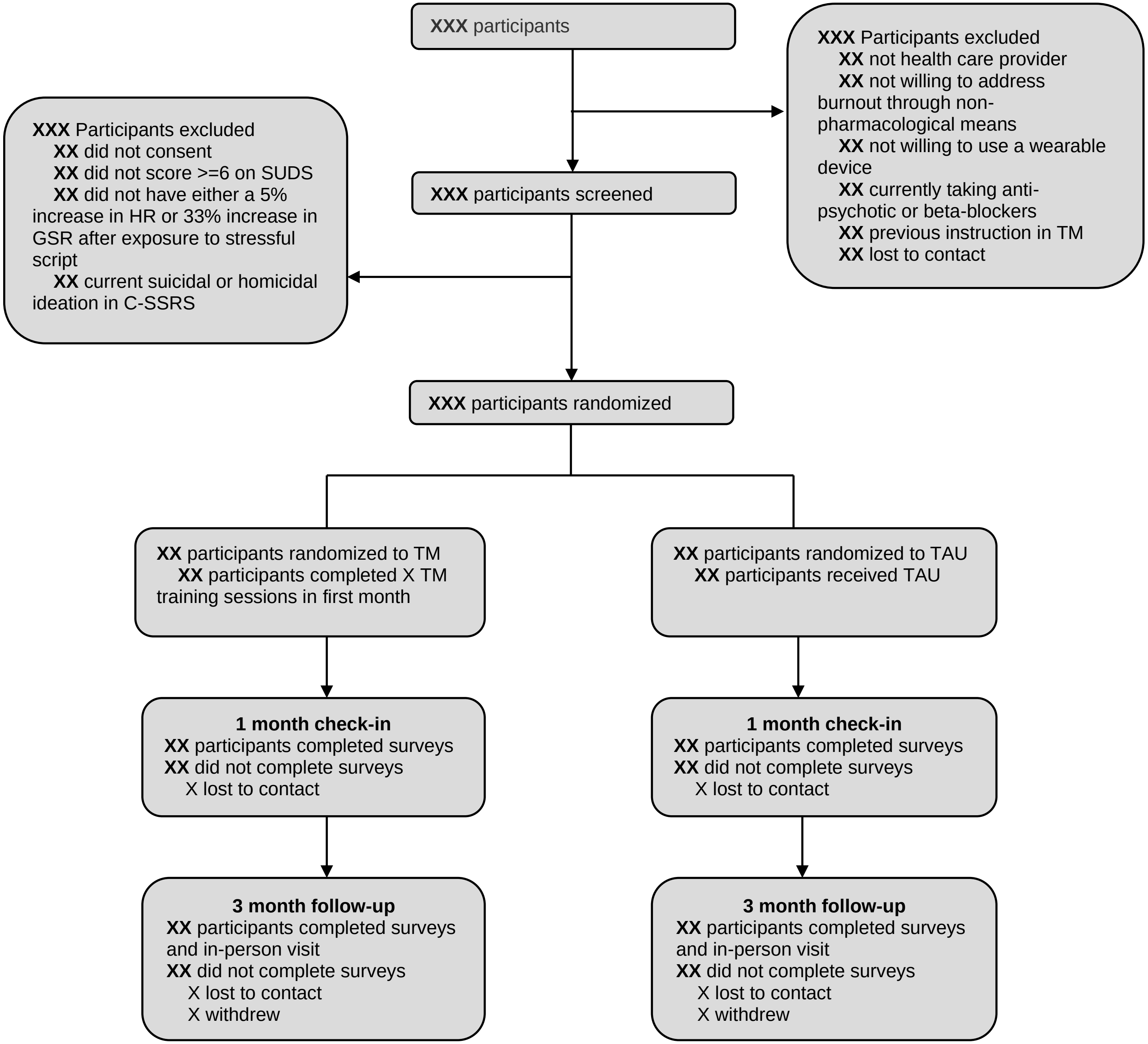

Table 1: Participant characteristics at baseline

Characteristics	TM (n=)	TAU (n=)	Total (n=)
Demographics			
Age<br>Median (Q1, Q3)			
Gender			
BMI			
Race<br>White/Caucasian<br>Black or African American<br>Asian<br>American Indian or Alaskan Native<br>Unknown or Not Reported			
Ethnicity<br>Hispanic<br>Non-Hispanic			
Marital status<br>Married/Partnered<br>Single/Separated/Divorced/Windowed			
Household size<br>1<br>2<br>3<br>4<br>5<br>6			
Medical History			
…			

Table 2: Patient survey endpoints at baseline and 3m

Endpoint	Baseline (all pats)	TM 3m (N=)	TAU 3m (N=)
BSI-18 Total Score			
BSI-18 Anxiety			
BSI-18 Depression			
BSI-18 Somatization			
MBI Total Score			
MBI Emotional Exhaustion			
MBI Depersonalization			
MBI Professional Accomplishment			
…			

Table 3: Effect size estimates

Endpoint	Effect estimate
BSI-18 Total Score	XX.XX
BSI-18 Anxiety	
BSI-18 Depression	
BSI-18 Somatization	
MBI Total Score	

MBI Emotional Exhaustion	
MBI Depersonalization	
MBI Professional Accomplishment	
…	

Table 4: GLM model fit estimates

Endpoint	TM change from baseline (95% CI)	TAU change from baseline (95% CI)	Mean Difference in Change from Baseline between Groups (95% CI)	p-value for interaction
BSI-18 Total Score				
BSI-18 Anxiety				
BSI-18 Depression				
BSI-18 Somatization				
MBI Total Score				
MBI Emotional Exhaustion				
MBI Depersonalization				
MBI Professional Accomplishment				
…				
