# Supplementary material for: Efficacy of Transcendental Meditation to Reduce Stress Among Health Care Workers: A Randomized Clinical Trial
Source: JAMA Netw Open. 2022 Sep 19;5(9):e2231917. doi: 10.1001/jamanetworkopen.2022.31917 (PMC9486450; doi:10.1001/jamanetworkopen.2022.31917)
Supplement: Supplement 2. — eMethods. Supplemental Code eTable 1. Inclusion and Exclusion Criteria eTable 2. Estimated Mean Changes Between Baseline and 1 Month in Transcendental Meditation and Control Groups eTable 3. Normalized GSI Score and BSI-18 Subscores at Baseline eTable 4. Estimated Mean Changes in Normalized GSI and BSI Subscores Between Baseline and 1 Month in Transcendental Meditation and Control Groups eFigure. Summary of Adherence Among Participants in the Transcendental Meditation Group [file jamanetwopen-e2231917-s002.pdf]

Supplementary Online Content

Joshi SP, Wong AKI, Brucker A, et al. Efficacy of Transcendental Meditation to reduce stress among health care workers: a randomized clinical trial. *JAMA Netw Open*. 2022;5(9):e2231917. doi:10.1001/jamanetworkopen.2022.31917

eMethods. Supplemental Code

eTable 1. Inclusion and Exclusion Criteria

eTable 2. Estimated Mean Changes Between Baseline and 1 Month in Transcendental Meditation and Control Groups

eTable 3. Normalized GSI Score and BSI-18 Subscores at Baseline

eTable 4. Estimated Mean Changes in Normalized GSI and BSI Subscores Between Baseline and 1 Month in Transcendental Meditation and Control Groups

eFigure. Summary of Adherence Among Participants in the Transcendental Meditation Group

This supplementary material has been provided by the authors to give readers additional information about their work.

© 2022 Joshi SP et al. *JAMA Network Open*.

eMethods. Supplemental Code

Below we present example SAS code (version 9.4, SAS Institute Inc) for the repeated measures analysis of the primary and secondary outcomes.

adat is an analytic dataset with outcomes captured in long (as opposed to wide) format. Each participant has multiple rows in ***adat*** corresponding to their measurements at each timepoint (0, 1, and 3). ***Record_id*** is a unique identifier for each participant. ***tm*** is a treatment indicator variable. ***t*** is a categorical variable taking values 0, 1, and 3 indicating the 3 timepoints. ***month1*** and ***month3*** are indicator variables with the following derivations: ***month1*** = ifelse(***t***==1, 1, 0); ***month3*** = ifelse(***t***==3, 1, 0). These indicators allow for non-linear time trends. The ***month1*tm*** and ***month3*tm*** terms allow for differential time trends between the TM and TAU arms after baseline. Finally, ***age_geq40*** and ***gender_f*** are randomization stratification factors which we adjust for in the analysis of the primary and secondary outcomes.

Correlation within participants is accounted for through the ***repeated*** statement. We specify an unstructured covariance structure (***type = un***) relating outcomes at the 3 timepoints within each participant.

```
proc mixed data=adat;
      class record_id t;
      model gsi = month1 month3 month1*tm month3*tm age_geq40 gender_f
/ solution ddfm = kenwardroger;
      repeated t / subject=record_id type=un rcorr;
      ods output Estimates=est1;
      estimate 'GSI: Difference of Differences in TM vs TAU: 3m vs
baseline' month3*tm 1 / cl;
      estimate 'GSI: TM 3m vs baseline' month3 1 month3*tm 1 / cl;
      estimate 'GSI: TAU 3m vs baseline' month3 1 / cl;
      run;
```

See Section 5.3 in Fitzmaurice, G. M., Laird, N. M., & Ware, J. H. (2012). Applied longitudinal analysis (Vol. 998). John Wiley & Sons for a presentation of the general linear models framework that corresponds to the SAS code above.

© 2022 Joshi SP et al. *JAMA Network Open*.

eTable 1. Inclusion and Exclusion Criteria

Inclusion Criteria	Exclusion Criteria
1. Full-time healthcare workers providing patient care currently during the COVID-19 pandemic (including physicians, trainee physicians, nurses, nurse practitioners, Physician Assistants, respiratory therapists, nurse assistants (NA), pharmacists, pharmacy technicians, occupational therapists, Radiology, neurology and cardiology clinical technicians/ technologist, Pulmonary Function technologists, Speech pathologists, Audiologists, Physical therapists, Exercise physiologists, Exercise specialist, Dietitian, Social worker, Patient health educator, Health Unit Coordinator, Nuclear med technician, Occupational Health technician, Certified medical assistant, Phlebotomy technician, Radiation therapist, Radio pharmacists, Vascular technician, Anesthesia technician, Behavior Health Technician, Cardiac sonographer, Podiatrist, Sonographer, Surgical technician, front desk staff, research/clinical healthcare workers without credentials, optometrists, emergency responders, EMS, dental assistants, orthodontists, endodontists, dentists, and dental hygienists.<br>2. A single-item stress scale will be used as a screen for eligibility; a minimum score of 6 on a 10-point response scale will be needed to meet inclusion criteria.<br>3. Subjects who have at least a 5% increase from baseline in heart rate after exposure to a personalized stressful script OR at least a 33% increase in skin conductance after exposure to the script.<br>4. Willingness to address burnout symptoms by non-pharmacological means.<br>5. All subjects must provide Informed Consent before enrollment in the study.<br>6. Willingness to wear the provided Apple Watch or Empatica device for the data collection process.	1. Anti-psychotic medications or beta-blockers<br>2. Current suicidal or homicidal ideation (suicidal ideation as screened by C-SSRI survey pre-enrollment). If positive, subjects will be sent to PCP or health care providers.<br>3. Previous instruction in TM

© 2022 Joshi SP et al. *JAMA Network Open*.

eTable 2. Estimated Mean Changes Between Baseline and 1 Month in Transcendental Meditation and Control Groups

eTable 2 shows estimates of within-group changes and between-group differences in outcomes between 1 month and baseline. These results are obtained from a repeated measures analysis with details provided in the main manuscript.

	**Estimated mean change between baseline and 1 month (95% CI)**			
**Outcome**	**TM**	**TAU**	**TM vs TAU**	**p-value for TM vs TAU**
GSI[a]	-3.8 (-5.6, -2.1)	-2.9 (-4.9, -0.9)	-0.9 (-3.3, 1.5)	0.44
BSI Somatization[a]	-0.6 (-1.2, -0.1)	-0.4 (-0.9, 0.2)	-0.3 (-1.0, 0.4)	0.43
BSI Depression[a]	-1.6 (-2.4, -0.8)	-0.7 (-1.6, 0.3)	-1.0 (-2.1, 0.2)	0.11
BSI Anxiety[a]	-1.8 (-2.6, -0.9)	-1.8 (-2.8, -0.8)	0.1 (-1.1, 1.3)	0.91
MBI Emotional Exhaustion[a]	-3.0 (-6.1, 0.1)	-0.3 (-4.0, 3.4)	-2.7 (-7.4, 2.0)	0.26
MBI Depersonalization[a]	-0.8 (-2.5, 0.8)	-0.3 (-2.3, 1.6)	-0.5 (-3.0, 2.0)	0.71
MBI Professional Accomplishment[b]	3.5 (1.9, 5.1)	2.1 (0.2, 4.1)	1.4 (-1.0, 3.8)	0.25
ISI[a]	-1.8 (-3.2, -0.3)	-0.6 (-2.3, 1.1)	-1.2 (-3.4, 1.1)	0.30
CD-RISC[b]	0.9 (-0.5, 2.4)	1.5 (-0.2, 3.2)	-0.6 (-2.8, 1.6)	0.60
PHQ-9[a]	-2.0 (-3.2, -0.8)	0.2 (-1.2, 1.6)	-2.2 (-3.9, -0.4)	0.02
GAD-7[a]	-2.2 (-3.4, -1.0)	-0.1 (-1.5, 1.3)	-2.1 (-3.8, -0.3)	0.02

Definition of abbreviations: GSI = Global Severity Index; BSI = Brief Symptom Inventory; MBI = Maslach Burnout Index; ISI = Insomnia Severity Index; CD-RISC = Conor-Davidson Resilience Scale; PHQ-9 = Patient Health Questionnaire-9; GAD-7 = General Anxiety Disorder-7; CI = confidence interval.

Ranges for the instruments are as follows: GSI (0 [best] to 72 [worst]), BSI subscales (0 [best] to 24 [worst]), MBI Emotional Exhaustion (0 [best] to 54 [worst]), MBI Depersonalization (0 [best] to 30 [worst]), MBI Professional Accomplishment (0 [worst] to 48 [best]), ISI (0 [best] to 28 [worst]), CD-RISC (0 [worst] to 40 [best]), PHQ-9 (0 [best] to 27 [worst]), GAD-7 (0 [best] to 21 [worst]).

Table S2 reports estimates of mean changes between baseline and 1 month within TM participants (column 1), within TAU participants (column 2), and differential changes in TM versus TAU participants (column 3). These estimates were obtained using a repeated measures modeling framework (see Methods section and code in Supplemental Material for complete model specification).

In this study, GSI was the primary outcome and all other scores are secondary outcomes.

[a] Negative estimates in these measures indicate improving outcomes between baseline and 1 month (columns 1 and 2) and greater improvements in the TM group compared to the TAU group (column 3).

[b] Positive estimates in these measures indicate improving outcomes between baseline and 1 month (columns 1 and 2) and greater improvements in the TM group compared to the TAU group (column 3).

© 2022 Joshi SP et al. *JAMA Network Open*.

eTable 3. Normalized GSI score and BSI-18 Subscores at Baseline

eTable 3 reports descriptive statistics on the normalized GSI and BSI-18 scores at baseline and at 3 months. Scores were normalized based on community norms by sex.

**Outcome**	**Baseline (all participants) N=80**	**TM at 3m N=40**	**TAU at 3m N=38**
Normalized GSI			
Mean (SD)	52.7 (8.8)	43.9 (8.2)	47.7 (10.6)
Median (25th, 75th)	54 (47, 59)	44 (38, 48)	48 (36, 55)
Min - Max	33 - 81	33 - 66	33 - 69
Normalized BSI Somatization			
Mean (SD)	48.4 (7.9)	44.3 (4.5)	47.8 (7.9)
Median (25th, 75th)	48 (41, 55)	42 (41, 48)	45 (41, 55)
Min - Max	41 - 74	41 - 55	41 - 68
Normalized BSI Depression			
Mean (SD)	52.4 (9.9)	46.4 (8.1)	49.5 (9.2)
Median (25th, 75th)	50 (45, 61)	45 (40, 49)	48 (40, 59)
Min - Max	40 - 81	40 - 74	40 - 70
Normalized BSI Anxiety			
Mean (SD)	53.9 (8.7)	45.3 (7.4)	48.4 (9.0)
Median (25th, 75th)	54 (48, 59)	45 (38, 50)	48 (38, 54)
Min - Max	38 - 81	38 - 65	38 - 69

Definition of abbreviations: GSI = Global Severity Index; BSI = Brief Symptom Inventory; SD = standard deviation.

Ranges for the instruments are as follows: Normalized GSI (33 [best] to 81 [worst]), BSI subscales (38 [best] to 81 [worst]).

© 2022 Joshi SP et al. *JAMA Network Open*.

eTable 4. Estimated Mean Changes in Normalized GSI and BSI Subscores Between Baseline and 1 Month in Transcendental Meditation and Control Groups

In eTable 4, we report estimates of within-group changes and between group-differences in normalized GSI and BSI-18 scores between baseline and 3 months. These results are obtained from a repeated measures analysis with details provided in the main manuscript.

Endpoint	Estimated mean change between baseline and 3 months (95% CI)			
**Outcome**	**TM**	**TAU**	**TM vs TAU**	**p-value for TM vs TAU**
Normalized GSI	-7.9 (-10.5, -5.3)	-5.9 (-8.5, -3.2)	-2.0 (-5.6, 1.6)	0.27
Normalized BSI Somatization	-3.2 (-5.0, -1.4)	-1.6 (-3.5, 0.3)	-1.6 (-3.9, 0.7)	0.18
Normalized BSI Depression	-5.3 (-8.0, -2.6)	-3.7 (-6.5, -1.0)	-1.5 (-5.0, 2.0)	0.39
Normalized BSI Anxiety	-8.4 (-10.9, -6.0)	-5.8 (-8.3, -3.2)	-2.7 (-5.9, 0.6)	0.10

Definition of abbreviations: GSI = Global Severity Index; BSI = Brief Symptom Inventory; SD = standard deviation.

Ranges for the instruments are as follows: Normalized GSI (33 [best] to 81 [worst]), BSI subscales (38 [best] to 81 [worst]).

© 2022 Joshi SP et al. *JAMA Network Open*.

eFigure. Summary of Adherence Among Participants in the Transcendental Meditation Group

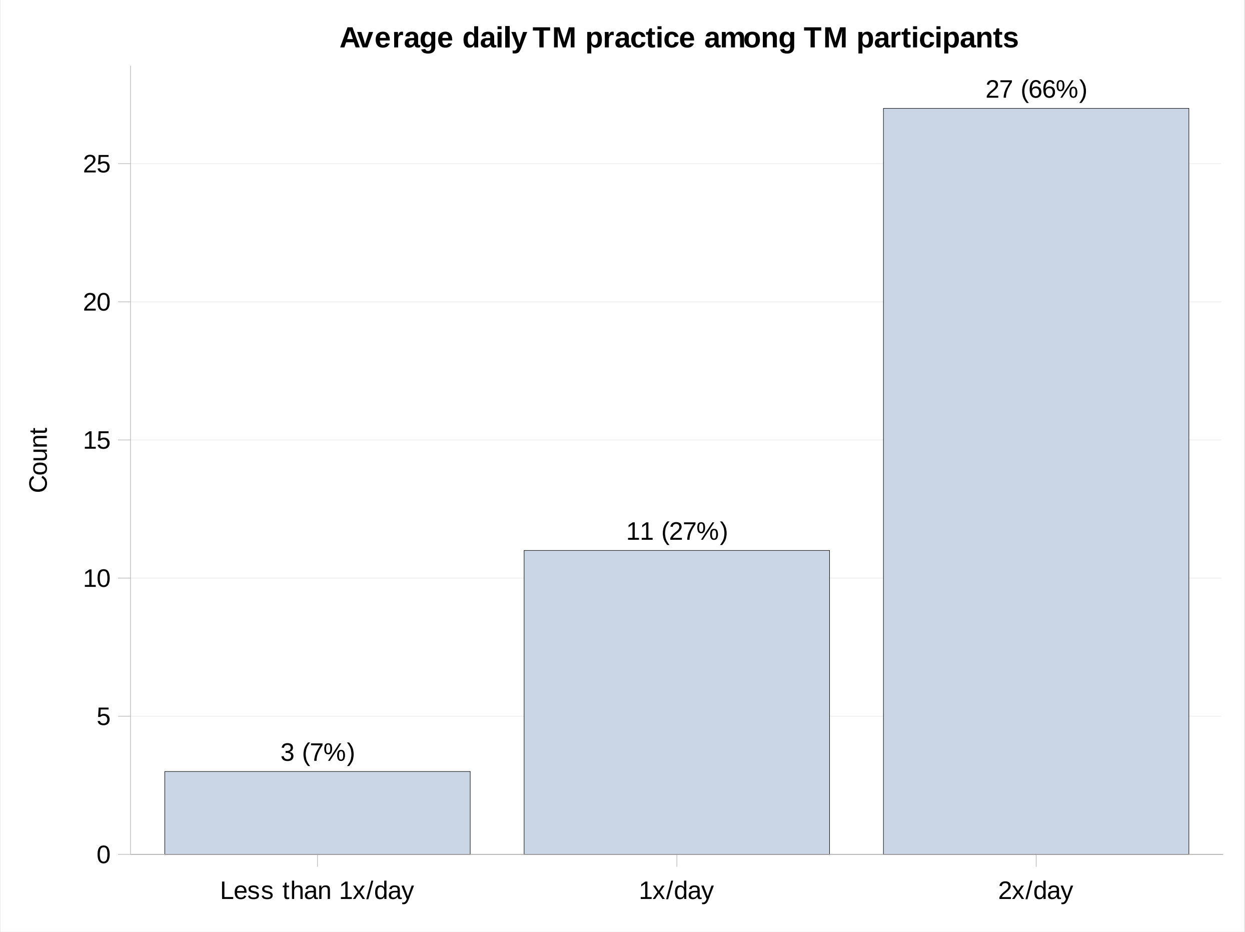

© 2022 Joshi SP et al. *JAMA Network Open*.
