# Supplementary material for: Efficacy of Transcendental Meditation to Reduce Stress Among Health Care Workers: A Randomized Clinical Trial
Source: JAMA Netw Open. 2022 Sep 19;5(9):e2231917. doi: 10.1001/jamanetworkopen.2022.31917 (PMC9486450; doi:10.1001/jamanetworkopen.2022.31917)
Supplement: Supplement 3. — Data Sharing Statement [file jamanetwopen-e2231917-s003.pdf]

Data Sharing Statement

Joshi. Efficacy of Transcendental Meditation to Reduce Stress Among Health Care Workers. *JAMA Netw Open*. Published September 19, 2022. doi:10.1001/jamanetworkopen.2022.31917

Data
Data available: Yes
Data types: Deidentified participant data, Data dictionary
How to access data: sangeeta.joshi@duke.edu
When available: With publication

Supporting Documents
Document types: None

Additional Information
Who can access the data: researchers whose proposed use of the data has been approved
Types of analyses: for Research purposes.
Mechanisms of data availability: after approval of a proposal, with a signed data access agreement
